# Supramolecular gels from sugar-linked triazole amphiphiles for drug entrapment and release for topical application†

**DOI:** 10.1039/c9ra02868d

**Published:** 2019-06-27

**Authors:** Komal Sharma, Jojo P. Joseph, Adarsh Sahu, Narender Yadav, Mohit Tyagi, Ashmeet Singh, Asish Pal, K. P. Ravindranathan Kartha

**Affiliations:** Department of Medicinal Chemistry, National Institute of Pharmaceutical Education and Research (NIPER) S. A. S. Nagar Punjab-160062 India rkartha@niper.ac.in; Institute of Nano Science and Technology Phase 10, Sector 64 Mohali Punjab-160062 India apal@inst.ac.in http://www.twitter.com/pal_asish

## Abstract

A simple molecular framework obtained by cross-linking a hydrophobic chain with *S*,*S*- and *R*,*R*-tetritol by the copper-catalysed azide–alkyne cycloaddition reaction is found to serve as an excellent bioisostere for self-assembly. The hexadecyl-linked triazolyl tetritol composite spontaneously self-assembles in *n*-hepane and methanol to form hierarchical organogels. Microscopic analyses and X-ray diffraction studies demonstrate eventual formation of nanotubes through lamellar assembly of the amphiphiles. A rheological investigation shows solvent-dictated mechanical properties that obey power law behavior similar to other low molecular weight gelators (LMOGs). The gel network was then utilized for the entrapment of drugs *e.g.* ibuprofen and 5-fluorouracil, with tunable mechanical behaviour under applied stress. The differential release profiles of the drugs over a period of a few hours as a result of the relative spatio-temporal location in the supramolecular network can be utilized for topical formulations.

## Abstract 2

Spontaneous formation of hierarchical supramolecular gels show solvent dictated self-assembly and spatio-temporal positioning of drug molecules in the network accounting for differential release profiles for topical application.

## Graphical abstract

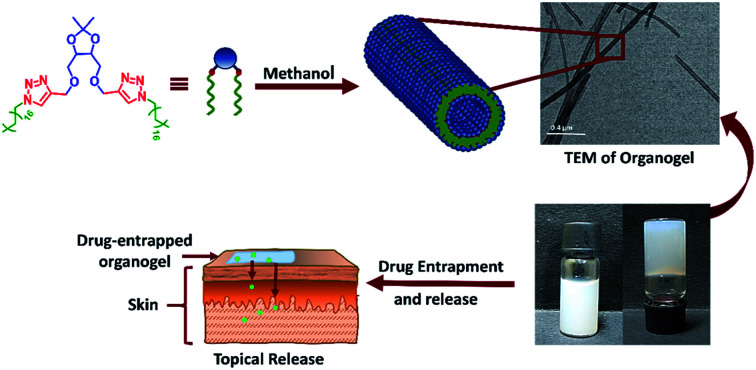

## Introduction

Self-assembly exists at both macroscopic and microscopic level in almost every aspect of nature, *e.g.*, arrangement of nucleotides in the double helical structure of DNA, formation of lipid tubules from phospholipids, generation of silk from monomeric silk fibroin, crystal growth by crystal engineering *etc.*^1^ Molecular self-assemblies, formed by spontaneous aggregation of molecules under thermodynamic equilibrium conditions, are mediated by a number of non-covalent interactions such as hydrogen bonds, ionic bonds, metal–ligand interactions, π–π stacking, electrostatic forces, hydrophobic forces and strong dipole–dipole associations and pave the way to realizing the formation of molecular crystals, colloids, nanofibrils, nanotubes, phase separated polymers, self-assembled monolayers and cross-linked gels in a bottom-up approach.^2^ Self-assembled fibrillar network (SAFIN)-mediated formation of low molecular weight gelators (LMOGs) employs a wide class of compounds from carbohydrates,^3^ and amino acid derivatives,^4^ to organo-metal complexes^5^*etc.* resulting in a number of interesting applications such as drug delivery and release,^6^ gene delivery,^7^ biomineralization,^8^ anion binding,^9^ tissue engineering, regenerative medicine,^10^ and oil spill recovery.^3*d*–*e*,11^ Controlled and sustained release of a drug meditated by LMOGs for dermal or transdermal application is an excellent approach in drug delivery, which requires local administration and circumvents specific side effects occurred in oral delivery and subsequent metabolism process.^12^ In such topical formulations involving organogel with entrapped drug, the organic media being antimicrobial and moisture insensitive, plays a crucial role.^13^

Carbohydrates are versatile building blocks for self-assembly as they encompass simple sugar units to complex granular starch-structure-like organization of (1,4)-α-linked glucans. Among the carbohydrate-based low molecular weight gelators, dialkanoate derivatives of mannitol and sorbitol have found interesting application in combating marine oil spills^3*d*,*e*,11,14^ and self-healing mediated soft optical devices.^15^ Triazole-linked carbohydrate-based self-assembling materials are envisaged as an interesting class of materials owing to the antimicrobial, antiviral and antitumor effects of the 1,2,3-triazole moiety.^16*a*^ Moreover, its structural features such as polarity, rigidity and ability to act as both hydrogen bond donor and acceptors make it a relevant bioisostere for designing a number of biologically important molecules *e.g.* glycerotriazolophanes and glycotriazololipids.^16^ Cu(i)-assisted azide–alkyne cycloaddition reaction (click reaction) has been used widely in the synthesis of several structures of varying complexities in the area of synthetic carbohydrate chemistry *e.g.* glycomimetics, glycodendrimers, glycorotaxanes *etc.*^17^

**Scheme 1:**
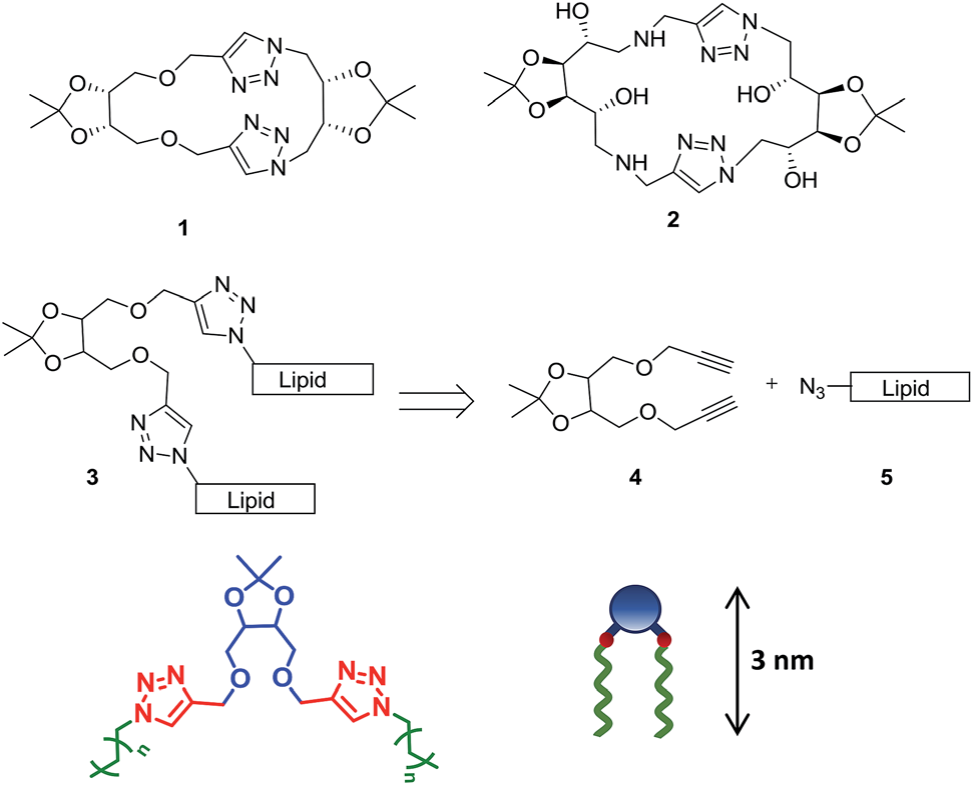
Rationale of designing acyclic tetritol-based lipids from triazolophanes and their general chemical structure along with the cartoon representation.

The glycerotriazolophanes with triazole ring-substituted carbohydrates, are highly metabolically stable as compared to the unsubstituted ones and possess the ability of π-stacking interaction thereby, facilitating the formation of self-assembled structures.^18^ However, the cyclic glycerotriazolophanes, 1–2 (Scheme 1) have rotational and flexibility constraints which may severely affect its solubility in various solvents. However, in its acyclic analogue, the flexibility of the lipid chains and the presence of the triazole ring make the molecule amphiphilic in nature, thereby widening its applications in designing artificial molecular receptors, chemical sensors, drug carrier systems, enzyme inhibitors *etc.*^19^ We postulated design of acyclic structures with tetritol unit, 3 as the platform to anchor the alkyne residue (4) for click chemistry with a fatty acid-based alkyl azide moiety (5).

Thus, we report the design of a new class of tetritol-based triazole-linked acyclic lipid derivatives and their self-assembly behaviour in different solvents to form supramolecular network and phase-selective gel. The structural features of the hierarchical self-assembled network starting from lamellar assembly of the building blocks to nanotubes and physically cross-linked network were deciphered with microscopy and X-ray diffraction. Detailed mechanical properties and flow behaviour of the gels were investigated to establish the thixotropic nature of the gel. The gelators, owing to the amphiphilic nature and network structures, were utilized to entrap both hydrophobic (ibuprofen) and hydrophilic (5-fluorouracil) drugs with the spatial selection, modulating the mechanical strength of the drug entrapped gel. The differential *in vitro* release profiles of the drugs spatially located in the self-assembled fibrillar network were investigated towards topical application.

## Experimental

### Gelation study

*R*,*R*- and *S*,*S*- tetritol-based lipid derivatives were dissolved in 1 mL of HPLC grade solvents *viz. n*-hexane, *n*-heptane, *n*-octane, methanol, dichloromethane, toluene, water and 1 : 1 mixtures of *n*-hexane/water and methanol/water in 2 mL glass vials with gradual heating. After complete dissolution, solutions were allowed to remain undisturbed at room temperature for 10 min to obtain the organogels.

### Microscopic analysis

The gels (0.7% w/v) were carefully scooped onto the carbon tapes on the aluminium stubs and were allowed to freeze dry. The dried gels were coated with gold for 20 seconds under high vacuum using BAL-TEC SSD-500 sputter coater instrument. Finally the morphology of the gels was imaged on a Hitachi S3400N SEM operated at 10 kV.

Diluted gel samples (0.35% w/v) were placed on a silicon wafer and were allowed to dry in air overnight. AFM Images of the dried samples were recorded with scan rate of 1 Hz in tapping mode analysis on a Bruker Multimode 8 scanning probe microscope with Bruker made antimony doped silicon cantilever of resonance frequency of 300 kHz and spring constant of 40 N m^−1^.

5 μL aliquot of the diluted gel fiber (0.02% w/v) in *n*-hexane/*n*-heptane/*n*-octane/methanol was drop-casted on a clean copper grid followed by room temperature drying. The samples were observed under TECNAI G^2^ F-20 high resolution transmission electron microscopy (HR-TEM) from FEI.

### XRD analysis

The organogels in *n*-heptane were heated to sol, and 100 μL of the sols were individually transferred carefully on to a pre-cleaned glass slide and left to air dry for 8 h to form the self-supported cast films on which measurements were performed using a Model-D8 Advance X-ray diffractometer. The X-ray beam generated with a Cu anode at the wavelength of K_α1_ beam at 1.5418 Å was directed toward the film edge, and scanning was done unto a 2*θ* value of 22°. Data were analysed and interpreted in terms of higher order reflections.

### Rheological studies

Rheological studies were performed on an Anton Paar MCR 302 rheometer with an adjustable Peltier temperature controlling system using a cone and plate geometry of CP-50-1. The gap distance between the cone and the plate was fixed at 0.1 mm. Oscillatory frequency sweep experiments were performed at constant amplitude of 0.05% strain which corresponds to the linear viscoelastic region of gel samples for an angular frequency range 200 to 0.001 rad s^−1^ at 20 °C. Stress amplitude sweep experiments were performed at a constant oscillation frequency of 10 rad s^−1^ for the strain range 0.001 to 100 at 20 °C. Temperature ramp measurements were performed at a constant oscillation frequency of 10 rad s^−1^ and constant oscillation amplitude of 0.05% strain for a temperature range of 20 to 50 °C at a heating and cooling rate of 5 °C min^−1^.

### Drug entrapment study & release study

Drug-entrapped gel was prepared by simultaneously dissolving the gelator (0.7%, w/v) and drug (ibuprofen or 5-fluorouracil) (1.5%, w/v) in 1 mL of methanol and heating it to 50 °C. The fluorescence emission spectra of ibuprofen and ibuprofen-entrapped gel/sol were obtained in the range of 270–400 nm upon excitation at 263 nm with excitation and emission slit width of 5 nm and 10 nm, respectively. The fluorescence emission spectra of 5-fluorouracil and 5-fluorouracil entrapped gel/sol were obtained in the range of 250–600 nm upon excitation at 230 nm with excitation and emission slit width of 5 nm each by using CARY-Eclipse fluorescence spectrophotometer, Varian. The fluorescence emission spectra and relative enhancement was compared to ascertain the entrapment of drug in the gel.

Entrapment efficiency helps in determining the amount of drug that has actually been entrapped inside the gel matrix. It was determined by centrifuging the drug-entrapped gel at 10 000 rpm for 5 min so that any free drug present comes up in the supernatant while the entrapped drug remain at the bottom within the gel. The collected supernatant was analyzed by HPLC to determine the drug content. The entrapment efficiency was then calculated by the formula:% EE = [(total amount of drug − amount of free drug)/total amount of drug] × 100

*In vitro* drug release behavior of the drugs entrapped in gel was determined by the dialysis bag method. The study was done on methanolic gel loaded with 0.5% w/v drug. The drug-entrapped gel was loaded in a dialysis bag suspended in a dissolution media comprising 50 mL water or phosphate buffer saline of pH 7.4. The dissolution media was kept at 37 °C with stirring at 100 rpm for 24–36 h. Aliquots of 1 mL each from the dissolution media were withdrawn at predetermined time intervals each time being replaced by an equal volume of fresh dissolution media (in order to keep the volume unchanged). The samples were analyzed using HPLC analytical technique.

## Result & discussion

**Scheme 2:**
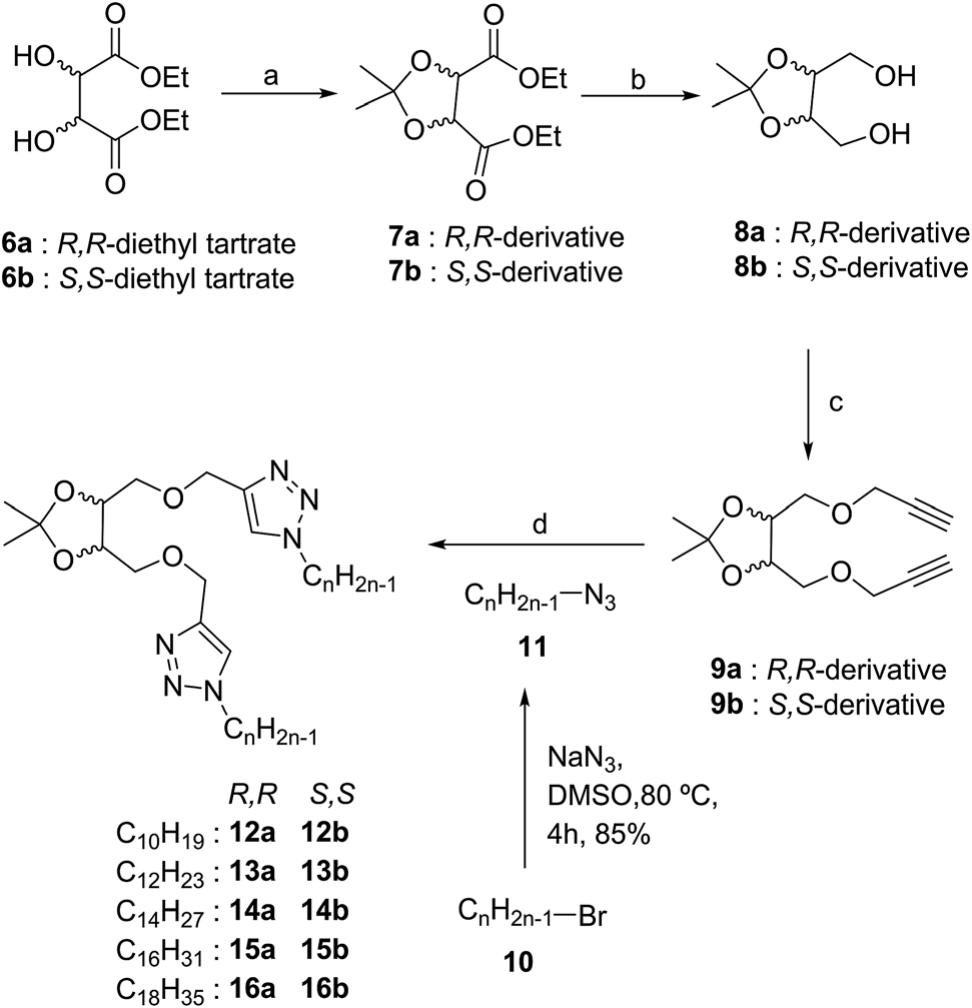
Synthesis of triazolyl lipids (12–16) cross-linked with tetritol (a) I_2_, acetone, rt, 12 h, 77%; (b) NaBH_4_, THF, 70 °C, reflux, 4 h, 78%; (c) propargyl bromide, NaH, DMF, 0 °C to >rt, 1 h, 71%; (d) CuSO_4_, sodium ascorbate, Ball mill (PM 100), 450 rpm, 85%.

Multistep synthesis of tetritol-based triazole-linked lipid derivatives was achieved using the Cu(i)-assisted azide–alkyne cycloaddition (CuAAC) reaction with *S*,*S*- and *R*,*R*-diethyl tartrate (6a and 6b) as the starting materials. The protection of the diol residues was performed with acetone in the presence of molecular iodine to obtain the desired isopropylidene acetal derivatives (7a/b). The acetonide-protected ester (7a/b) was then reduced with NaBH_4_ and the derived 1,4-diol (8a/b) was then propargylated with propargyl bromide in the presence of NaH to obtain the dipropargyl ether 9a/b (Scheme 2). A series of alkyl azides, 11 were prepared from the commercially available bromides 10 by a standard bimolecular nucleophilic substitution and were reacted with 9a/b under solvent-free click reaction conditions by grinding in a ball mill. The 1,4-di-*O*-triazolylated tetritol derivatives (12a/b–16a/b) having carbon chain length varying from C10 to C18, were obtained in good yields with improved solubility in CH_2_Cl_2_, CHCl_3_ and toluene (unlike the rather insoluble triazolophanes 1 and 2). The ^1^H NMR spectrum of the compound exhibited a singlet of two protons at *δ* 7.58 ppm, characteristics of the two triazolyl hydrogens (one on each of the two triazole units) in the structure (*cf.* ESI†).

The self-assembly of the compounds 12–16 was investigated by dissolving them in the desired solvent by warming to obtain a saturated solution which upon gradual cooling led to the formation of certain loosely assembled structural units *via* stochastic nucleation, further interactions among which resulting in tightly packed gels. The formation of gel was ascertained by the inverted vial method, in which a perfect gel immobilise all the solvent such a way that it does not ooze out on inversion of the vial (*cf.* ESI, Fig. S1†). The properties of gels depend on the nature of the solvent, amphiphilicity and the concentration of the gelator. Compounds 12–16 were tested for gelation in non-polar solvents, *e.g. n*-hexane, *n*-heptane, octane, toluene and dichloromethane, in polar solvents, *e.g.* methanol, ethanol, dimethyl sulfoxide (DMSO) and water, and mixtures of solvents, *i.e. n*-heptane/water and methanol/water (Table 1). For the series of molecules in non-polar solvents, an increase in the hydrophobic lipid chain length (from C10 to C18) was observed to lead to a gradual change in the gelation tendency. Thus, while an aggregate or weak gel was obtained for 12a/b, a stable gel was obtained for 15a/b (with low mgc values, 0.21%, w/v), whereas the octadecyl-linked molecules (16a/b) gave a precipitate, whereby indicating the requirement of an optimally tuned hydrophilic/hydrophobic balance in the compounds for the desired gel to be formed. On the other hand, in polar solvents such as methanol, ethanol and DMSO, opaque gels were obtained with 15a/b and 16a/b as the gelator (mgc 0.3–0.5%, w/v). However, these compounds remained insoluble in water and dissolved completely in dichloromethane and toluene even at room temperature, thus, forming a clear solution. A study of the gelation property of these compounds in various solvents using a gelator concentration in the range 0.2–1% w/v manifested similar self-assembly behaviour for the *S*,*S*-derivatives to their corresponding *R*,*R*-analogues. The gels exhibited reversible gel-to-sol transition on repeated heating (in the range of 40–45 °C) and cooling using the inverted vial method. However, the gels from methanol were found to have slightly higher gel-to-sol transition temperature as compared to that from *n*-heptane.

**Table 1:** Gelation ability of tetritol-based triazole-linked lipid derivatives 12–16 in different solvents at 25 °C^a^

Entry	*n*-Hexane	*n*-Heptane	*n*-Octane	MeOH	EtOH	DMSO	CH_2_Cl_2_ & toluene
12a	A	A	A	S	S	A	S
13a	A	A	A	S	S	A	S
14a	A	A	A	S	S	W	S
15a	G (0.25)	G (0.21)	G (0.20)	G (0.34)	G (0.40)	G (0.30)	S
16a	P	P	P	G (0.30)	G (0.45)	G (0.36)	S
12b	A	A	A	S	S	A	S
13b	W	W	W	S	S	W	S
14b	G (0.72)	G (0.62)	G (0.66)	S	S	W	S
15b	G (0.32)	G (0.25)	G (0.21)	G (0.38)	G (0.42)	G (0.34)	S
16b	P	P	P	G (0.35)	G (0.50)	G (0.38)	S

a A (aggregate), G (gel), P (precipitate), S (solution) and W (weak gel) denote the result of dissolution of the particular compound in the solvent indicated; the values in parenthesis show the minimum gelator concentration (mgc) in % w/v.

**Fig. 1:**
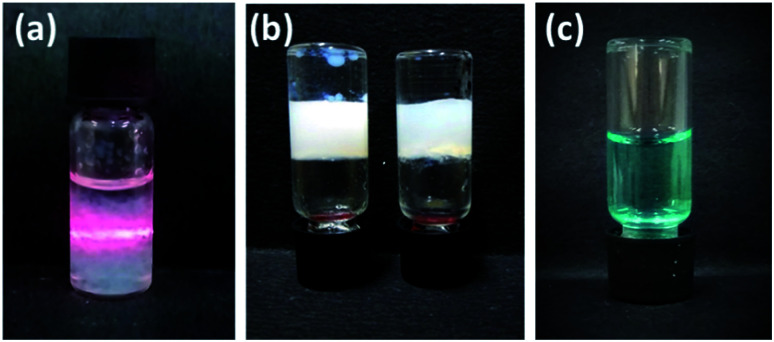
(a) Tyndall scattering from the colloidal suspension of 0.1%, w/v of 15a in *n*-heptane. (b) Phase selective gelation by 15a (0.7% w/v gelator) in a mixture of *n*-heptane/*n*-hexane and water in 1 : 1 ratio. (c) Metal-responsive gel-to-sol transition for the gel of 15a in methanol on adding Cu^2+^ ions (0.02% w/v).

Tyndall effect typical of particles of the colloidal range *i.e.* 40 nm to 1 μm was also observed for these molecules (Fig. 1a). When the laser beam was passed through 0.1% w/v gelator in *n*-heptane, scattering of the beam was observed as the self-assembled structures grew to attain particle size of colloidal range (Fig. 1a). Next, the phase selective gelation behaviour was investigated using a mixture of immiscible solvents, *n*-heptane and water. Compound 15a selectively gelated the *n*-heptane layer of the binary mixture of the solvents (Fig. 1b). The free triazole ring played an important role in the gelation process. In order to check the stimuli-responsive nature of the gel network, we resorted to the use of different metal ions that binds with the trizole rings as such interactions are efficiently exploited in applications *e.g.* chemosensing, biological evaluation and selective trapping of metal ions. Upon adding metal ions, *e.g.* Cu^2+^ and Zn^2+^, the gel network broke down to yield clear solution (Fig. 1c) indicating the metal-responsive nature of the gel presumably due to the ligation of the respective metal ions to the triazole rings in the molecule.

**Fig. 2:**
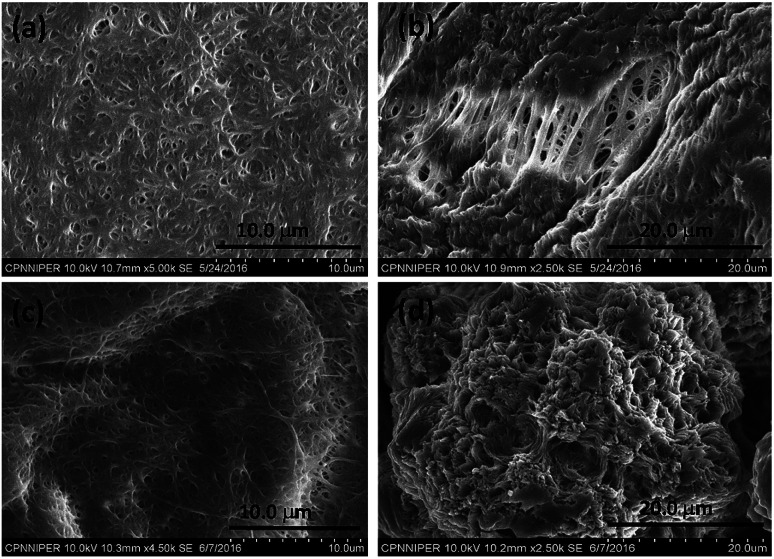
Scanning electron micrograph of compound 15a (0.7%, w/v) in (a) *n*-heptane (b) methanol; 15b in (c) *n*-heptane (d) methanol.

**Fig. 3:**
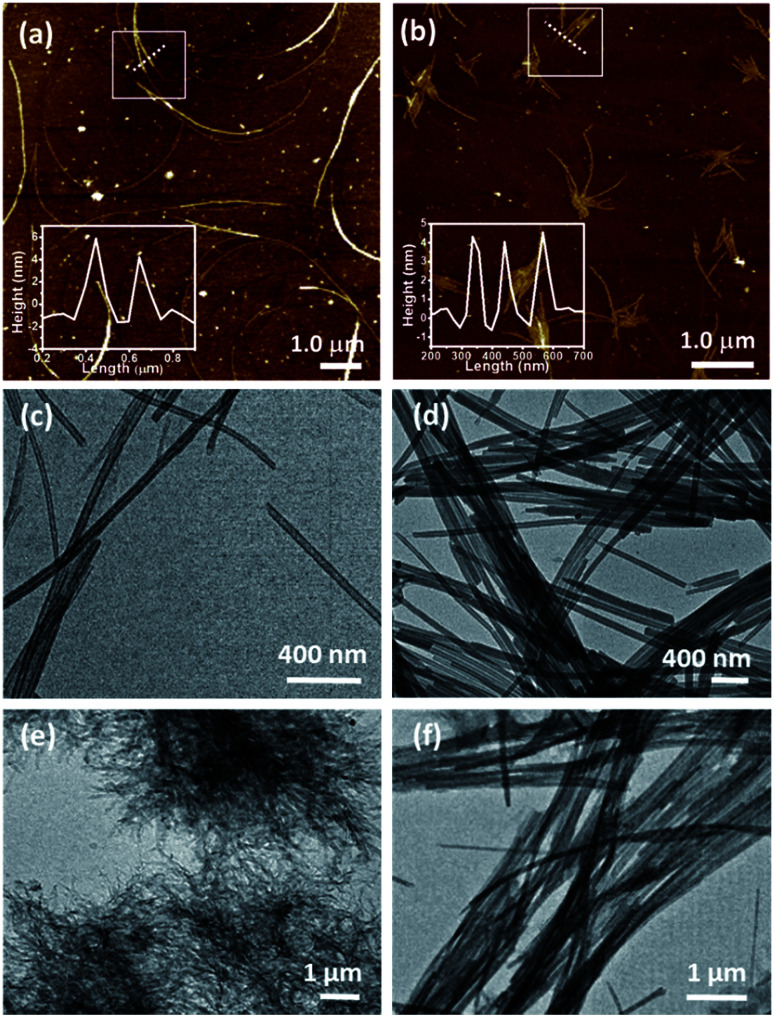
Atomic Force micrograph of tetritol-anchored triazolyl lipid 15a in (a) *n*-heptane and (b) methanol, respectively. Transmission electron micrographs of 15a depicting the assembly into bundles of molecular straw in (c) *n*-heptane and (d) methanol. (e) and (f) demonstrate complete disruption and restoration of the tubular nanostructures in hot methanol and at 25 °C, respectively.

The hexadecyl-linked tetritol derivatives 15a–b were chosen for further studies as they formed gels readily in almost all the organic solvents examined. To discern the nature of the microstructures and morphologies present in the gels, we thoroughly examined the gel samples of 15a–b by using different microscopic techniques *viz.* SEM, TEM and AFM. Representative SEM of the dried gel of 15a–b obtained from *n*-heptane and methanol showed the presence of three dimensional networks of fibers with varying thickness and porous structures that could entrap solvent molecules (Fig. 2). Atomic force microscopic studies on the diluted gels (0.35%, w/v, from *n*-heptane) obtained from 15a revealed long fibres of high aspect ratio with the height of 6–7 nm (Fig. 3a). On the other hand the gel from methanol consisted of relatively short fibres with 5–6 nm in height that aggregated to form mesh-like clusters (Fig. 3b). AFM recorded for diluted gel samples also showed a presence of polydispersed nanoparticles, as a result of initial self-assembly of gelators, which might act as nucleation sites for 1-dimensional growth of fibers with eventual formation of three dimensional gel network.^20^ The difference in the morphologies in polar and non-polar solvents might be due to the existence of stochastic non-covalent interactions between the gelator and the solvent molecules, paving way for a competition between solvent–gelator and solvent–solvent interactions. TEM images obtained for the diluted gel (0.02%, w/v) of 15a from *n*-heptane and methanol showed the inner structures of the fibres, which were found to be hollow and thus straw-like in appearance (Fig. 3c and d). The long nanotubes (“straws”, length ∼ 5 μm) from both *n*-heptane and methanol had the outer and the inner diameters of 45 ± 5 nm and 24 ± 3 nm respectively with shell thickness of 10 ± 3 nm. This indicates spontaneous self-organization in the presence of the solvents to form distinct molecular straw-like tubular arrangement. The effect of temperature was observed on the methanolic gel formed by 15a by subjecting it to sequential heating and cooling cycles followed by microscopic analysis at each of the stages. The gel on heating dissolves completely and forms a clear solution with TEM images indicating a complete disruption of the straw structure with the resultant formation of clusters of short elongated assembled structures (Fig. 3e). However, the methanol solution on standing at ambient temperature (25 °C) for a few minutes, led to complete restoration of the straw pattern (Fig. 3f), thereby demonstrating the reversible nature of the assembly.

**Fig. 4:**
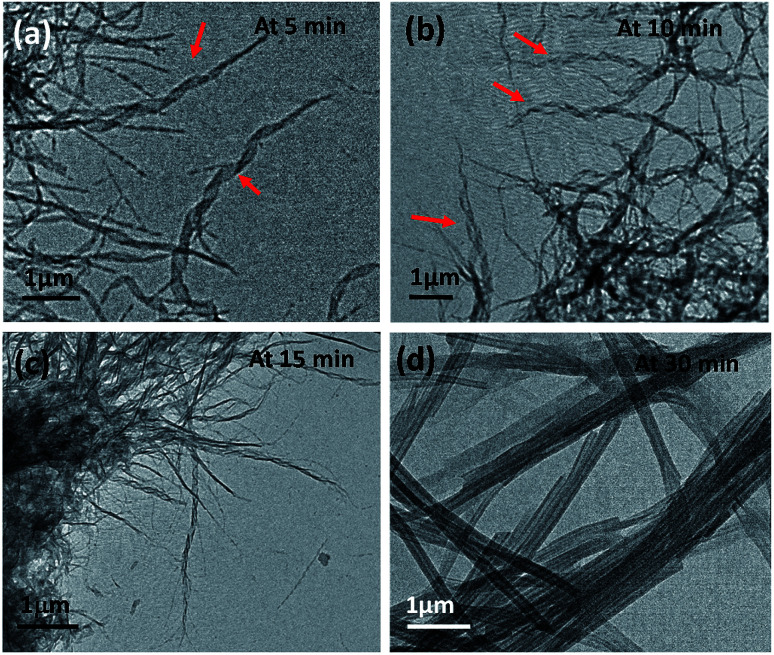
TEM showing time-dependent gel formation by 15a in methanol after heating it to a sol followed by incubation at 25 °C for (a) 5 min, (b) 10 min, (c) 15 min and (d) 30 min.

Time-dependent TEM images were recorded by heating a gel to sol followed by gradual cooling to ambient temperature till complete immobilization of the solvent occurred. The micrographs obtained at different time intervals *viz.* 5 min, 10 min, 15 min and 30 min demonstrate the formation of intermediate structures (Fig. 4a–d). Initially, the molecules of the gelator assemble themselves to give a thin helical ribbon-like structure, which undergoes further intertwining. The finely intertwined ribbons on further incubation condense to give compact molecular straws. Each turn in the helical ribbon comes closer to the previous turn by non-covalent interactions thereby, leading to stacking over one another to form a tube like hollow structure.^21^ Further, an examination of the assembly from 15a at the initial stages, or in a dichloromethane solution, showed the presence of right-handed turns in the ribbon which can be compared with the origami structures made using the palm leaves (*cf.* ESI, Fig S2†). Indeed, the analogous *S*,*S*-configured 15b did exhibit the matching left-handed turns in the structure formed in CH_2_Cl_2_, which suggests that even though the assembly is not strong enough to manifest in gel structures in such solvents, ensembles with distinct patterns of distinct shapes and dimensions, or properties, can be formed in the process of attaining stable ordered arrangements by adapting to the environment suitably.^22*a*^ Additionally, in this particular class of self-assembled structures the molecular chirality can be extended to the net supramolecular chirality as confirmed from the mirror image of the CD band in the wavelength of 215 to 260 nm for the diluted gels of 15a–b (*cf.* ESI, Fig. S3†).^22*b*–*c*^

**Fig. 5:**
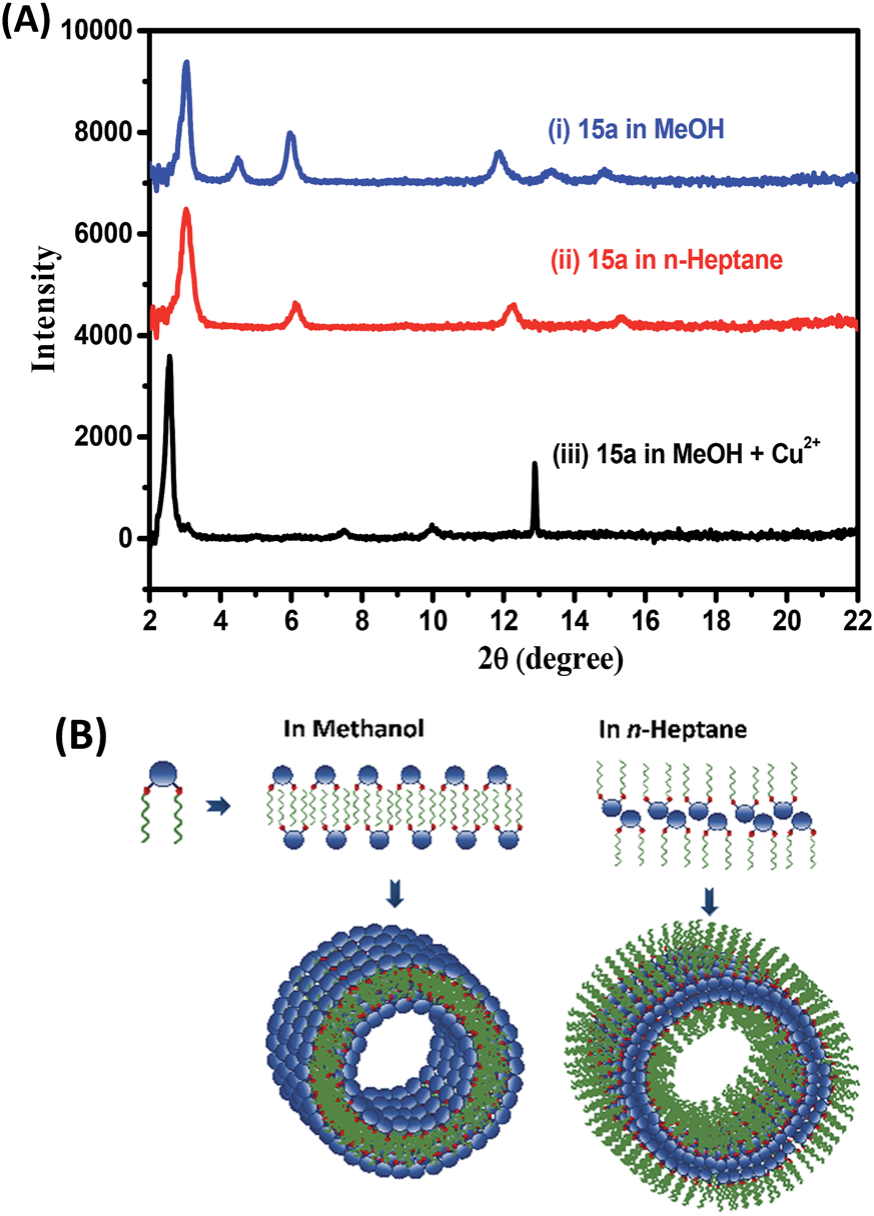
(A) X-ray diffraction intensities obtained for the gel formed by 15a in (i) methanol and (ii) *n*-heptane; and (iii) the methanol gel of 15a with Cu^2+^ ions in 1 : 1 ratio (B) possible assembly-pattern leading to the formation of nanotubes in methanol and in *n*-heptane.

The X-ray diffraction pattern of the cast film from the gel of 15a in methanol and *n*-heptane showed periodical diffraction peaks, indicating an ordered layered structure. The gel formed in methanol and *n*-heptane as in Fig. 5A(i–ii) and Table S1† showed a predominantly intense peak at 2*θ* = 2.97 that corresponds to periodicity of 2.9 nm in the structures of the aggregates. This matches well with the extended length of the molecules (3 nm) in their energy-minimized structures. We propose a multiple lamellar bilayer of the amphiphiles that folds into nanotubes. However, solvents such as *n*-heptane and methanol differ in polarity and the effective gelator–solvent interactions mediated by chain interdigitation. Hence, the solvents dictate the nature and the extent of exposure of the hydrophilic head groups and hydrophobic tails (Fig. 5B). The assembly obtained from methanol solution of 15a and Cu^2+^ ions in 1 : 1 ratio showed an intense peak at 2*θ* = 2.5 corresponding to a periodicity of 3.46 nm in the structures of the aggregates as in Fig. 5A(iii). It suggests significant metal–triazole ligand complexation that in turn disrupts the packing and increases the dimension of the bilayer assembly. This must also be accompanied by a net change in the overall polarity of the assembled particles eventually resulting in the observed dissolution of the gel structure (Fig. 1c).

**Fig. 6:**
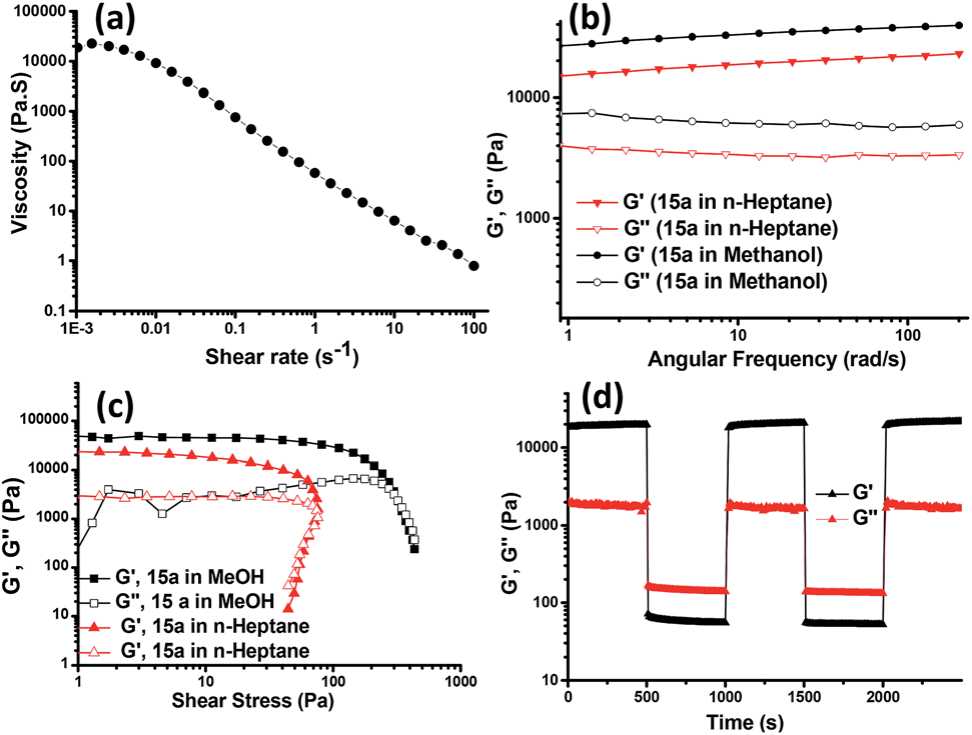
(a) Typical flow curve measurement of methanol gels 15a. (b) Typical oscillatory frequency sweep and (c) oscillatory amplitude sweep measurement for the gel made in methanol and *n*-heptane with 15a (1% w/v). (d) Thixotropic behaviour by oscillatory rheology for three cycles with strain variation from 0.05% to 100% in periodic manner for gel prepared from 15a in MeOH.

Rheological studies were then carried out to gather information on the mechanical strength and the flow behaviour of the 15a-derived gels in *n*-heptane and methanol. The rotational flow curve experiment showed zero shear viscosity of ∼20 000 Pa s^−1^, which on applying increasing shear rate exhibited the shear-thinning behaviour of the gel with a scaling decay, *η* ∝ (d*γ*/d*t*)^−0.97^ and a negative slope approaching −1, indicating the viscoelastic solid-like property of the gel (Fig. 6a). In an oscillatory frequency sweep experiment for strong viscoelastic solid-like gels, the storage (*G*′) moduli are usually a magnitude higher as compared to the loss (*G*′′) moduli. The gels from 15a in methanol and *n*-heptane showed a frequency-independent behaviour for *G*′ and *G*′′ over two decades of the angular frequency with a *G*′/*G*′′ value of 5.8 (Fig. 6b). The oscillatory amplitude sweep showed (Fig. 6c) a linear viscoelastic region (LVR) with *G*′ > *G*′′ till a critical stress beyond which the gel started to flow. The values for the corresponding stress, known as yield stress (*σ*_y_), indicated a high degree of robustness of the gels. The gel made in methanol had slightly higher value of *G*′ and *G*′′ as compared to the gel obtained in *n*-heptane, hence, proving the methanolic gel to be stronger. Also, the gel obtained in DMSO was found to possess a *G*′ value of an order higher than that of the gels in ethanol and methanol (*cf.* ESI, Fig. S4A†). To verify the power law behavior of the gel, (*G*′∝ *c*^*m*^), the logarithmic plot of storage modulus of the gel *vs.* the gelator concentration was used to obtain the value of *m* (Fig. S4B†). The yield stress (*σ*_y_) also increased with the gelator concentration, and scaled as the power of the gelator concentration (*σ*_y_ ∝ *c*^*m*^). The values of the exponents (*m* and *n*) are 2.26 and 2.90, respectively, which are in good agreement with the reported values for low molecular mass gelling agents.^23^

Thixotropic nature of the organogel was investigated by applying alternating 0.05% and 100% strain for three cycles, which exhibited instantaneous self-recovery of the network after the removal of high strain. Real time temperature ramp study with constant angular frequency (10 rad s^−1^) and 0.05% strain for the gel made in methanol showed gel-to-sol transition with abrupt decrease in *G*′ upon heating and sol-to-gel transformation during the cooling cycle, with recovery in *G*′ value (see ESI, Fig. S4C†) suggesting reformation of the gel structures which is in agreement with the temperature dependent TEM investigation. The applied shear led to the intermittent disruption of the straw structure as evident from the TEM images and upon removal of the shear stress the assembly recovered to its original straw structure (Fig. 4).

**Fig. 7:**
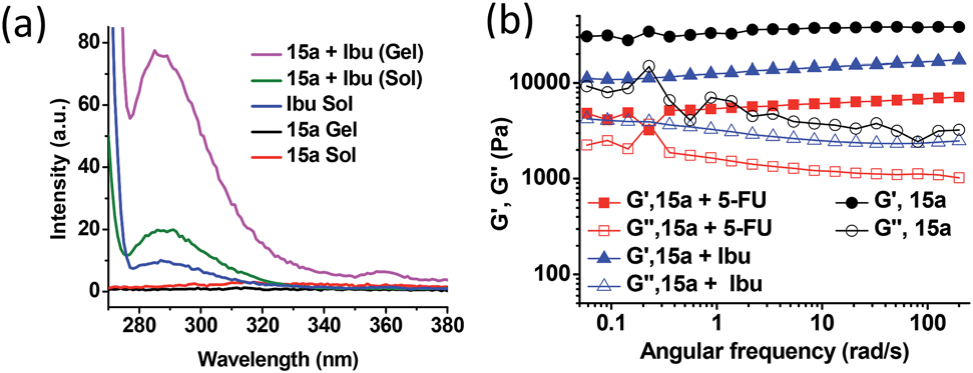
(a) Fluorescence emission spectra of ibuprofen in both gel and sol state. (b) Typical oscillatory frequency sweep for the methanol gels of 15a (1%, w/v) without any drug (control) and with of 5-FU (0.36% w/v) and Ibu (0.45% w/v).

In view of the amphiphilic nature of the compounds described above, we explored the possibility of entrapping both hydrophobic and hydrophilic drugs in the gel network. Ibuprofen (Ibu) and 5-fluorouracil (5-FU) were chosen as the model hydrophobic and hydrophilic drugs, respectively, because of their relatively small size, contrasting polarity attributes and their applications as topical agents. Effective and successful transdermal delivery of such drugs circumvent possible side effects *e.g.* gastric irritation, mouth sores, ulcers, diarrhoea, nausea and heartburn. The fluorescence emission spectra of the two drugs were recorded individually and in the presence of the methanol gel of 15a, in both sol and the gel state. The fluorescence of the hydrophobic drug, Ibu increased nearly 2-fold in the presence of the gelator in the sol state at 50 °C and further to approximately 8-fold on cooling it to the gel state at 25 °C (Fig. 7a). Such increases in the fluorescence suggest effective entrapment of the drug in the hydrophobic pockets of the self-assembled structures mediated by the van der Waal's interaction among the hydrophobic chains.^24^ However, for the hydrophilic drug, 5-FU a fluorescence intensity enhancement of only 3-fold was observed in the gel state relative to that in sol, suggesting localization of the drug towards relatively hydrophilic exterior of the gelators engaging in hydrogen bonding interaction with the trizole moieties of the gelator (see ESI, Fig. S5A†). The entrapment efficiency of Ibu and 5-FU as determined from the HPLC peak area calculation, was found out to be 0.45% and 0.36% w/v with respect to the gelator and solvent, respectively, indicating reasonably good extent of drug loading in the self-assembled network. The oscillatory frequency sweep for the native gel of 15a and the gel containing 5-FU (0.36%, w/v) and Ibu (0.45%, w/v) drugs in methanol suggests a decrease in the *G*′ values for the drug-loaded organogel network as compared to the native gel (Fig. 7b). Also, the deviation from the frequency-independent behaviour for the drug-loaded organogels suggests relatively weaker organogel strength due to incorporation of the drug molecules. However, Ibu-loaded organogel did not show as much decrease in *G*′ values as the 5-FU-loaded gel. This is presumably due to the weaken interaction among the conformationally rigid polar head group due to the loading of 5-FU, while the Ibu can be loaded in relatively flexible long chain interior without compromising much of the interactions. The yield stress beyond which the gel starts to flow shows remarkable decrease in the case of the 5-FU-loaded gel as compared to that of the Ibu-loaded one (see ESI, Fig. S5B†).

**Fig. 8:**
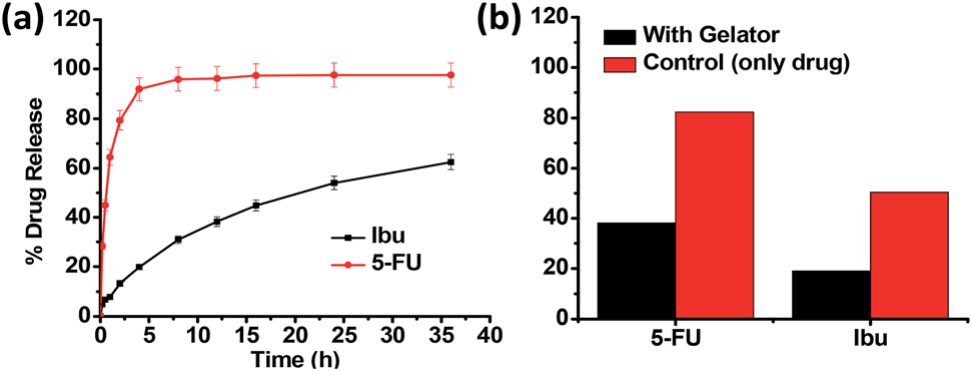
Drug release profile for ibuprofen and 5-fluorouracil in (a) water at 37 °C. (b) Histogram profiles for the release of the drugs encapsulated in the methanol gel of 15a and in methanol as control using water as the dissolution media after 1 h.

The *in vitro* drug release behaviour of the drugs entrapped in the gel network was subsequently examined by the method of dialysis. From the drug release profile of Ibu and 5-FU (Fig. 8a), we observed that the maximum cumulative release of 5-FU up to 95% occurred within the first 8 h of the study when water was used as the dissolution media, however, the Ibu release was relatively slower, and after 36 h only 62% of the drug was found released. This corroborate the relative positioning of the drugs in the hydrophilic exterior/hydrophobic interior, respectively, and their subsequent release to (and the dissolution in) the dissolution media. Upon changing the dissolution media (from water) to phosphate buffer (pH 7.4), the release of Ibu took place much faster, with 94% release occurring in 8 h, compared to the 30% release observed in water, owing to its higher solubility and intrinsic dissolution rate in the buffer media mediated by higher pH and inclusion of salt in dissolution medium (see ESI, Fig. S6†).^25^ Thus, it can be concluded that using the gels derived from 15a, drugs of varying hydrophilic/lipophilic balance could be incorporated in their structural cavities with varying resultant drug release characteristics. Fig. 8b shows the effect of the three-dimensional network of the gel on the release pattern for both Ibu and 5-FU in comparison with the control (that is, without the presence of a gelator). In the absence of the gelator the drug release was comparatively faster in both the drugs. Thus, it was observed that after 1 h of the study, 5-FU release was only 38% instead of the nearly 80% release observed in the absence of the gelator. Likewise, in the case of Ibu also the extent of the drug release observed was significantly lower (18%) in the presence of the gelator as against the nearly 50% value obtained in its absence.

## Conclusion

In summary, we have developed a new class of *S*,*S*-/*R*,*R*-tetritol-based triazole-linked lipid derivatives as successful low molecular weight organogelators suitable for a number of organic solvents and biphasic solvent-mixtures. The self-assembly pattern obtained by using microscopic techniques has revealed generation of nanotubes in methanol and *n*-heptane *via* gradual structural transformations. Rheological analyses addressed the mechanical properties and self-healing nature of the native gels as dictated by the solvent–gelator interaction. Further, the amphiphilic domains of the nanostructures with differential interaction with the drug molecules was efficiently utilised as a distinct templates for entrapment and controlled release of both hydrophobic (ibuprofen) and hydrophilic (5-fluorouracil) drugs. The spatial location of the drugs in the amphiphilic domains modulate the mechanical strength of the drug-entrapped gel based on the drug–gelator interactions. Additionally, the gels can be utilized for small molecular drug delivery platform and the release profile of the drugs can efficiently be fine-tuned using the knowledge of spatial location and interactions among the drug–gelator combinations. Such rational correlations of structural and mechanical information of the drug-entrapped gel network is a crucial addition in the repertoire of gel-mediated drug entrapment and dual drug release study for topical administration in future.

## Conflicts of interest

There are no conflicts to declare.

## Supplementary Material

RA-009-C9RA02868D-s001
