# Supplementary material for: Supramolecular gels from sugar-linked triazole amphiphiles for drug entrapment and release for topical application
Source: RSC Adv. 2019 Jun 27;9(34):19819–27. doi: 10.1039/c9ra02868d (PMC9065371; doi:10.1039/c9ra02868d)

Electronic Supplementary Material (ESI) for RSC Advances.
This journal is © The Royal Society of Chemistry 2019

Supramolecular Gels from Sugar-linked Triazole Amphiphiles for Drug Entrapment and Release for Topical Application

Komal Sharma[a], Jojo Joseph[b], Adarsh Sahu[a], Narender Yadav[a], Mohit Tyagi[a], Ashmeet Singh[b], Asish Pal[b]* and K. P. Ravindranathan Kartha[a]*

[a] *Department of Medicinal Chemistry, National Institute of Pharmaceutical Education and Research (NIPER), S. A. S. Nagar, Punjab-160062, India. Email: rkartha@niper.ac.in*

[b] *Institute of Nano Science and Technology, Phase 10, Sector 64, Mohali, Punjab-160062, India. Email: apal@inst.ac.in*

Table of Contents

1.	Materials and Methods	S-2
2.	Synthesis and Characterizations	S-2
3.	Gelation study	S-9
4.	Microscopic characterization	S-10
5.	Circular Dichroism Spectra	S-11
6.	XRD analysis	S-12
7.	Rheology	S-13
8.	Drug entrapment study	S-14
9.	In-vitro drug release study	S-14
10.	References	S-15
11.	NMR Spectra	S-16

1. Materials & Methods:

R, R - and *S, S* -diethyl tartrate, molecular iodine, sodium borohydride, propargyl bromide, sodium hydride, copper sulphate and sodium ascorbate were purchased from Sigma-Aldrich Chemicals Ltd. 5-Fluoro uracil and racemic ibuprofen were obtained from Sigma. Solvents used for the chemical synthesis acquired from commercial sources were of analytical grade and were used without further purification unless otherwise stated. Solvents for reactions were dried by standard procedures and stored over activated molecular sieves (3Å). Reactions were monitored by TLC, which was performed with 0.2 mm Merck pre-coated silica gel 60 F_{254} Aluminium sheets. Compounds were detected by charring i.e. dipping the TLC plates in an ethanolic solution of phosphomolybdic acid (5% v/v) and heating. NMR spectra were recorded on Bruker Advance DPX (400 MHz) spectrometer. Chemical shift are reported in parts per million (ppm) units relative to tetramethylsilane (TMS) as internal standard. Coupling constant (*J*) are reported in Hertz (Hz).

2. Synthesis & Characterizations

2.1. Procedure for synthesis of Diethyl-2,3-*O*-isopropylidene-*R, R*-tartrate (7a)[1]

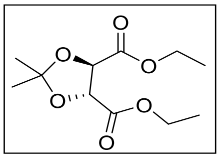

R, R -Diethyl tartrate (10 g, 48.5 mmol) was added to anhydrous acetone (500 mL) in 1 lit round bottom flask then, iodine (1.29 g, 11.8 mmol) was added to it. The reaction mixture was stirred at room temperature for 3 h. Completion of the reaction was monitored by TLC in 2:1 eluent, after which, aqueous solution of sodium thiosulphate was added to the reaction mixture till it become colorless. The reaction mixture was then concentrated to 1/3rd of its original volume and was diluted with CH_2Cl_2. The organic layer was washed successively with $NaHCO_3$ solution and water. It was then dried over anhydrous Na_2SO_4 and concentrated under reduce pressure. Pure compound **7a** was isolated using column chromatography. Yield: 77%; yellowish liquid; ^{1}H NMR (400 MHz, $CDCl_3$) δ 4.62-4.60 (1H, m, C**H**OC(CH_3)$_2$), 4.18-4.09 (2H, m, OC**H**$_2$CH$_3$), 1.41-1.33 (3H, m, OC(C**H**$_3$)$_2$), 1.23-1.18 (3H, t, OCH$_2$C**H**$_3$); ^{13}C NMR (100 MHz, $CDCl_3$) δ 169.41, 113.50, 61.54, 26.18, 13.87; HRMS: *m/z* calculated for $C_{11}H_{18}O_6$: 269.10 $[M+Na]^+$ Found: 269.0993 $[M+Na]^+$.

2.2. Diethyl-2,3-*O*-isopropylidene-*S,S*-tartrate (7b)

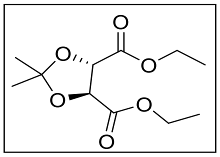

S,S -Diethyl tartrate (10 g, 48.5 mmol) was added to anhydrous acetone (500 mL) in 1 L round bottom flask then, iodine (1.29 g, 11.8 mmol) was added to it. Further procedure is same as that of compound **7a**. Yield: 80%; yellowish liquid; ^{1}H NMR (400 MHz, $CDCl_3$) δ 4.77 (1H, s, C**H**OC(CH_3)$_2$), 4.30-4.25 (2H, m, OC**H**$_2$CH$_3$), 1.50 (3H, s, OC(C**H**$_3$)$_2$), 1.33-1.30 (3H, m, OCH$_2$C**H**$_3$); ^{13}C NMR (100 MHz, $CDCl_3$) δ 169.67, 113.75, 61.90, 26.36, 14.09; LCQ: *m/z* calculated for $C_{11}H_{18}O_6$: 269.10 [M+Na]$^+$ Found: 269.12 [M+Na]$^+$

2.3. Procedure for synthesis of 2,3-*O*-isopropylidene- *R, R* -tetritol (8a)[2]

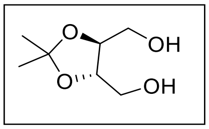

Compound **7a** (4.11 g, 16.7 mmol) was weighed in a 2 necked round bottom flask and THF (30 mL) was added to it. After that $NaBH_4$ (4 g, 100.5 mmol) was added over a period of 15 min in small fractions and the reaction mixture was refluxed at 70 °C. Then MeOH (15 mL) was added to it dropwise over a period of 20 min. The progress of reaction was monitored by TLC. When the substrate was completely consumed i.e. after 4 h, the reaction mixture was cooled down to room temperature and saturated solution of NH_4Cl (20 mL) was added to it. The reaction was completely quenched with the appearance of clear solution. After that the solvent in the reaction mixture was completely dried and the crude product was obtained by washing the solid residue with CH_2Cl_2 and filtering it through sintered funnel. The organic layer was dried over Na_2SO_4 and concentrated to give crude product which was then purified by silica gel column chromatography. Compound **8a** was obtained as slightly yellowish liquid in 92% yield; ^{1}H NMR (400 MHz, $CDCl_3$) δ 3.99-3.97 (1H, t, C**H**OC(CH_3)$_2$), 3.79-3.69 (2H, m, C**H**$_2$OH), 2.98 (1H, br, CH$_2$O**H**), 1.42 (3H, s, OC(C**H**$_3$)$_2$); ^{13}C NMR (100 MHz, $CDCl_3$) δ 109.31, 78.20, 62.08, 26.98; HRMS: *m/z* calculated for $C_7H_{14}O_4$: 185.0789 [M+Na]$^+$ Found: 185.0788 [M+Na]$^+$

2.4. 2,3-*O*-Isopropylidene- *S,S* -tetritol (8b)

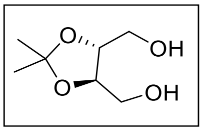

Compound **7b** (5 g, 20.3 mmol) was weighed in a 2 necked round bottom flask and THF (30 mL) was added to it with subsequent addition of $NaBH_4$ (4.6 g, 122.8 mmol) over a period of 15 min in small fractions. Further procedure is same as that of compound **8a**. Yield: 94%; yellowish liquid; ^{1}H NMR (400 MHz, $CDCl_3$) δ 3.97

(1H, t, C**H**OC(CH_3)$_2$), 3.79-3.60 (2H, m, C**H**$_2$OH), 3.13 (1H, br, CH_2O**H**), 1.42 (3H, s, OC(C**H**$_3$)$_2$); ^{13}C NMR (100 MHz, $CDCl_3$) δ 109.29, 78.30, 62.58, 62.12, 26.73, 26.99; LTQ: *m/z* calculated for $C_7H_{14}O_4$: 185.0789 $[M+Na]^+$ Found: 184.98 $[M+Na]^+$

2.7. Procedure for synthesis of 2,3-*O*-isopropylidene-1,4-*O*-dipropargyl- *R, R* -tetritol (9a)[3]

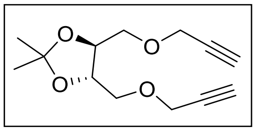

Compound **8a** (2 g, 12.3 mmol) was dissolved in anhydrous DMF (50 mL) in a 100 mL round bottom flask, at 0 ºC, NaH (1.8 g, 37.5 mmol) was added portion wise with continuous stirring. After 15 min propargyl bromide (4 g, 3.23 mol) was added to it. The reaction was monitored by TLC. After completion of reaction (1 h), 3.5 mL of MeOH was added to the reaction mixture. The solvent was evaporated under reduced pressure and diluted with CH_2Cl_2 and washed with water. The organic layer was dried over Na_2SO_4 and concentrated to give crude product which was then purified by silica gel column chromatography. Yield: 85%; yellowish liquid; ^{1}H NMR (400 MHz, $CDCl_3$) δ 4.23-4.22 (2H, d, C**H**$_2$CCH), 4.03-4.01 (1H, m, C**H**OC(CH_3)$_2$), 3.72-3.64 (2H, m, C**H**$_2$OCH$_2$CCH), 2.44 (1H, t, CH_2C≡C**H**), 1.42 (3H, s, OC(C**H**$_3$)$_2$); ^{13}C NMR (100 MHz, $CDCl_3$) δ 109.89, 79.3, 74.83, 70.20, 58.73, 26.96; MS LCQ: *m/z* calculated for $C_{13}H_{18}O_4$: 260.1102 $[M+Na]^+$ Found: 260.89 $[M+Na]^+$

2.8. 2,3-*O*-Isopropylidene-1,4-*O*-dipropargyl- *S,S* -tetritol (9b)

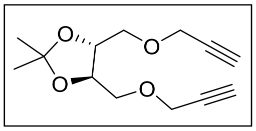

Compound **8b** (3.5 g, 21.58 mmol) was dissolved in anhydrous DMF (50 mL) in a 100 mL round bottom flask, at 0 ºC, NaH (1.8 g, 37.5 mmol) was added portion wise with continuous stirring. After 15 min propargyl bromide (7.7 g, 7.7 mol) was added to it. Further procedure for the synthesis of compound **9b** is same as that of compound **9a**. Yield: 85%; yellowish liquid; ^{1}H NMR (400 MHz, $CDCl_3$) δ 4.25-4.24 (2H, m, C**H**$_2$CCH), 4.05-4.03 (1H, m, C**H**OC(CH_3)$_2$), 3.74-3.66 (2H, m, C**H**$_2$OCH$_2$CCH), 2.46 (1H, t, alkynic-H), 1.46 (3H, s, OC(C**H**$_3$)$_2$); ^{13}C NMR (100 MHz, $CDCl_3$) δ 109.93, 79.29, 74.86, 70.21, 58.75, 26.97; MS LTQ: *m/z* calculated for $C_{13}H_{18}O_4$: 260.1102 $[M+Na]^+$ Found: 261.04 $[M+Na]^+$

2.9. General procedure for synthesis of various triazole derivatives

Di-*O*-propargyl derivatives (**9a/9b**) (1 mmol) and alkyl azide (**11**) (2 mmol) were taken in stainless steel cup containing 10 stainless steel balls (10 mm diameter). Further $CuSO_4.7H_2O$ (0.4 mmol) and sodium ascorbate (0.8 mmol) were added to it. The reaction mixture was allowed to grind in

PM-100 ball mill at 450 rpm for 6-7 h. The reaction was continuously monitored by TLC using 3:5 eluent. After the completion of the reaction the reaction mixture was dissolved in CH_2Cl_2 and MeOH. The solvent was evaporated and the crude reaction mixture was purified by column chromatography.

(i) (2*R*,3*R*)-2,3-*O*-Isopropylidene-1,4-bis-((1-decyltriazol-4-yl)methoxy)butane (12a)

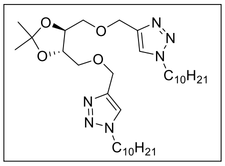

Compound **9a** (500 mg, 2.09 mmol), 1-azidodecane (776 mg, 4.17 mmol), $CuSO_4$ (209 mg, 0.84 mmol), sodium ascorbate (333 mg, 1.67 mmol) were taken in 40 mL stainless steel cup and rest of the procedure was followed as mentioned above. 1.142 g; 90%; colourless solid; 40 ºC; $[\alpha]_D$: -6.5 (*c* 1,$CHCl_3$); ^{1}H NMR (400 MHz, $CDCl_3$) δ 7.55 (1H, s, triazole-**H**), 4.67 (2H, q, *J* = 16.16 Hz, OC**H**$_2$C$_2$HN$_3$), 4.32 (2H, t, *J* = 7.28 Hz, C**H**$_2$(CH$_2$)$_8$CH$_3$), 3.95 (1H, t, *J* = 2.08 Hz C**H**OC(CH$_3$)$_2$), 3.64 (2H, dd, J_1 = 3.12 Hz, J_2 = 3 Hz, C**H**$_2$CHOC(CH$_3$)$_2$), 1.87 (2H, t, *J* = 6.92 Hz, CH$_2$C**H**$_2$(CH$_2$)$_7$CH$_3$), 1.42 (3H, s, CHOC(C**H**$_3$)$_2$), 1.41-1.26 (22H, bm, CH$_2$(C**H**$_2$)$_8$CH$_3$), 0.86 (3H, t, *J* = 6.56 Hz, CH$_2$(CH$_2$)$_8$C**H**$_3$); ^{13}C NMR (100 MHz, $CDCl_3$) δ 144.77, 122.39, 109.78, 77.29, 71.00, 65.06, 50.37, 31.89, 30.30, 29.68, 29.59, 29.51, 29.38, 29.32, 29.00, 26.98, 26.51, 22.67, 14.09; MS LCQ: *m/z* calculated for $C_{33}H_{60}N_6O_4$: [M] 604.47 Found: [M] 604.62.

(ii) (2*S*,3*S*)-2,3-*O*-Isopropylidene-1,4-bis-((1-decyltriazol-4-yl)methoxy)butane (12b)

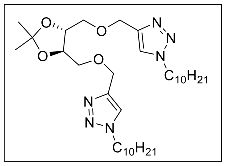

Compound **9b** (400 mg, 1.68 mmol), 1-azidodecane (338 mg, 1.846 mmol), $CuSO_4$ (167 mg, 0.67 mmol), Sodium ascorbate (265 mg, 1.34 mmol) were taken in 40 mL stainless steel cup and rest of the procedure was followed as mentioned above. 0.915 g; yield: 88%; colourless solid; 48 ºC; $[\alpha]_D$: 6.5 (*c* 1,$CHCl_3$); ^{1}H NMR (400 MHz, $CDCl_3$) δ 7.57 (1H, s, triazole-**H**), 4.71 (2H, q, *J* = 16.24 Hz, OC**H**$_2$C$_2$HN$_3$), 4.36 (2H, t, *J*= 7.36 Hz, C**H**$_2$(CH$_2$)$_8$CH$_3$), 4.00-3.99 (1H, m, C**H**OC(CH$_3$)$_2$), 3.62 (2H, dd, J_1 = 8.8 Hz, J_2 = 3.23 Hz, C**H**$_2$CHOC(CH$_3$)$_2$), 1.91 (2H, t, *J* = 6.96 Hz, CH$_2$C**H**$_2$(CH$_2$)$_7$CH$_3$), 1.42 (3H, s, CHOC(C**H**$_3$)$_2$), 1.36-1.27 (14H, bm, CH$_2$(C**H**$_2$)$_8$CH$_3$), 0.89 (3H, t, *J* = 6.64 Hz, CH$_2$(CH$_2$)$_8$C**H**$_3$); ^{13}C NMR (100 MHz, $CDCl_3$) δ 144.78, 122.41, 109.81, 77.29, 71.01, 65.07, 50.38, 31.85, 30.32, 29.48, 29.40, 29.25, 29.01, 27.00, 26.51, 22.66, 14.11; MS LTQ: *m/z* calculated for $C_{33}H_{60}N_6O_4$: $[M+Na]^+$ 627.45 Found: $[M+Na]^+$ 627.38.

(iii) (2*R*,3*R*)-2,3-*O*-Isopropylidene-1,4-bis-((1-dodecyltriazol-4-yl)methoxy)butane (13a)

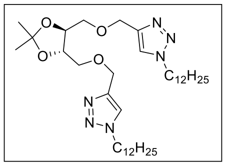

Compound **9a** (400 mg, 1.68 mmol), 1-azidododecane (710 mg, 3.35 mmol), $CuSO_4$ (167 mg, 0.67 mmol), sodium ascorbate (266 mg, 1.34 mmol) were taken in 40 mL stainless steel cup and rest of the procedure was followed as mentioned above. 0.953 g; yield: 86%; colourless solid; 45 ºC; $[\alpha]_D$: -5.4 (*c* 1,$CHCl_3$); ^{1}H NMR (400 MHz, $CDCl_3$) δ 7.57 (1H, s, triazole-**H**), 4.70 (2H, q, J = 16.08 Hz, OC**H**$_2C_2HN_3$), 4.35 (2H, t, J = 7.28 Hz, C**H**$_2(CH_2)_{10}CH_3$), 3.99-3.98 (1H, m, C**H**OC$(CH_3)_2$), 3.66 (2H, dd, J_1 = 3.48 Hz, J_2 = 3.04 Hz, C**H**$_2$CHOC$(CH_3)_2$), 1.91 (2H, t, J = 7 Hz, CH_2C**H**$_2(CH_2)_9CH_3$), 1.42 (3H, s, CHOC(C**H**$_3)_2$), 1.41-1.26 (22H, bm, CH_2(C**H**$_2)_{10}CH_3$), 0.89 (3H, t, J = 6.64 Hz, $CH_2(CH_2)_{10}$C**H**$_3$); ^{13}C NMR (100 MHz, $CDCl_3$) δ 144.77, 122.39, 109.78, 77.29, 71.00, 65.06, 50.37, 31.89, 30.30, 29.68, 29.59, 29.51, 29.38, 29.32, 29.00, 26.98, 26.51, 22.67, 14.09; MS LCQ: *m/z* calculated for $C_{37}H_{68}N_6O_4$: $[M+Na]^+$ 683.52 Found: $[M+Na]^+$ 683.37.

(iv) (2*S*,3*S*)-2,3-*O*-Isopropylidene-1,4-bis-((1-dodecyltriazol-4-yl)methoxy)butane (13b)

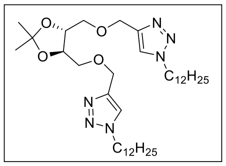

Compound **9b** (400 mg, 1.68 mmol), 1-Azidododecane (710 mg, 3.35 mmol), $CuSO_4$ (167 mg, 0.67 mmol), sodium ascorbate (265 mg, 1.34 mmol) were taken in 40 mL stainless steel cup and rest of the procedure was followed as mentioned above. 0.975 g; yield: 88%; colourless solid; 52 ºC; $[\alpha]_D$: 5.3 (*c* 1,$CHCl_3$); ^{1}H NMR (400 MHz, $CDCl_3$) δ 7.57 (1H, s, triazole-**H**), 4.70 (2H, q, J = 16.28 Hz, OC**H**$_2C_2HN_3$), 4.36 (2H, t, J = 3.88 Hz, C**H**$_2(CH_2)_{10}CH_3$), 4.00-3.98 (1H, m, C**H**OC$(CH_3)_2$), 3.67 (2H, dd, J_1 = 3.48 Hz, J_2 = 3.04 Hz, C**H**$_2$CHOC$(CH_3)_2$), 1.91 (2H, t, J = 7 Hz, CH_2C**H**$_2(CH_2)_9CH_3$), 1.42 (3H, s, CHOC(C**H**$_3)_2$), 1.33-1.26 (18H, bm, CH_2(C**H**$_2)_{10}CH_3$), 0.89 (3H, t, J = 6.64 Hz, $CH_2(CH_2)_{10}$C**H**$_3$); ^{13}C NMR (100 MHz, $CDCl_3$) δ 144.77, 122.41, 109.80, 77.29, 71.01, 65.07, 50.38, 31.90, 30.32, 29.60, 29.52, 29.40, 29.33, 29.01, 26.99, 26.52, 22.68, 14.09; MS LTQ: *m/z* calculated for $C_{37}H_{68}N_6O_4$: $[M+Na]^+$ 683.52 Found: $[M+Na]^+$ 683.49.

(v) (2*R*,3*R*)-2,3-*O*-Isopropylidene-1,4-bis-((1-tetradecyltriazol-4-yl)methoxy)butane (14a)

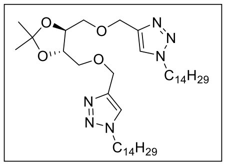

Compound **9a** (500 mg, 2.09 mmol), 1-azidotetradecane (1.00 g, 4.17 mmol), $CuSO_4$ (209 mg, 0.84 mmol), sodium ascorbate (333 mg, 1.67 mmol) were taken in 40 mL stainless steel cup and rest of the procedure was followed as mentioned above. 1.329 g; yield: 88%; colourless solid; 53 ºC; $[\alpha]_D$: -4.1 (*c* 1,$CHCl_3$); ^{1}H NMR (400 MHz, $CDCl_3$) δ 7.57 (1H, s, triazole-**H**), 4.71 (2H, q, J = 16.12 Hz, OC**H**$_2C_2HN_3$), 4.36 (2H,

t, J = 7.28 Hz, C**H**$_2$(CH$_2$)$_{12}$CH$_3$), 4.00-3.98 (1H, m, C**H**OC(CH$_3$)$_2$), 3.67 (2H, dd, J_1 = 2.12 Hz, J_2 = 1.6 Hz, C**H**$_2$CHOC(CH$_3$)$_2$), 1.91 (2H, t, J = 7 Hz, CH$_2$C**H**$_2$(CH$_2$)$_{11}$CH$_3$), 1.42 (3H, s, CHOC(C**H**$_3$)$_2$), 1.33-1.27 (22H, bm, CH$_2$(C**H**$_2$)$_{12}$CH$_3$), 0.89 (3H, t, J = 6.68 Hz, CH$_2$(CH$_2$)$_{12}$C**H**$_3$); ^{13}C NMR (100 MHz, CDCl$_3$) δ 122.38, 109.79, 71.01, 65.07, 50.37, 31.91, 30.31, 29.67, 29.63, 29.60, 29.52, 29.39, 29.34, 29.01, 26.99, 26.52, 22.68, 14.11; MS LCQ: *m/z* calculated for $C_{41}H_{76}N_6O_4$: [M+Na]$^+$ 739.58 Found: [M+Na]$^+$ 739.47.

(vi) (2*S*,3*S*)-2,3-*O*-Isopropylidene-1,4-bis-((1-tetradecyltriazol-4-yl)methoxy)butane (14b)

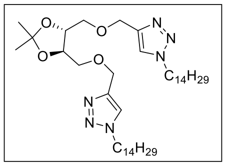

Compound **9b** (500 mg, 2.09 mmol), 1-azidotetradecane (1.00 g, 4.17 mmol), CuSO$_4$ (209 mg, 1.68 mmol), sodium ascorbate (333 mg, 0.84 mmol) were taken in 40 mL stainless steel cup and rest of the procedure was followed as mentioned above. 1.293 g; yield: 86%; colourless solid; 64 ºC; [α]$_D$: 6.1 (*c* 1,CHCl$_3$); ^{1}H NMR (400 MHz, CDCl$_3$) δ 7.56 (1H, s, triazole-**H**), 4.68 (2H, q, J = 16.36 Hz, OC**H**$_2$C$_2$HN$_3$), 4.34 (2H, t, J = 7.23 Hz, C**H**$_2$(CH$_2$)$_{12}$CH$_3$), 3.98-3.96 (1H, m, C**H**OC(CH$_3$)$_2$), 3.65-3.65 (2H, t, J_1 = 2 Hz, J_2 = 1.12 Hz, C**H**$_2$CHOC(CH$_3$)$_2$), 1.91-1.87 (2H, t, J = 6.96 Hz, CH$_2$C**H**$_2$(CH$_2$)$_{11}$CH$_3$), 1.39 (3H, s, CHOC(C**H**$_3$)$_2$), 1.31-1.24 (22H, bm, CH$_2$(C**H**$_2$)$_{12}$CH$_3$), 0.87 (3H, t, J = 6.6 Hz, CH$_2$(CH$_2$)$_{12}$C**H**$_3$); ^{13}C NMR (100 MHz, CDCl$_3$) δ 144.74, 122.42, 109.77, 77.27, 71.98, 65.03, 50.36, 31.90, 30.30, 29.66, 29.63, 29.59, 29.51, 29.39, 29.34, 29.00, 26.98, 26.50, 22.67, 14.11; MS LTQ: *m/z* calculated for $C_{41}H_{76}N_6O_4$: [M+Na]$^+$ 739.58 Found: [M+Na]$^+$ 739.59.

(vii) (2*R*,3*R*)-2,3-*O*-Isopropylidene-1,4-bis-((1-hexadecyltriazol-4-yl)methoxy)butane (15a)

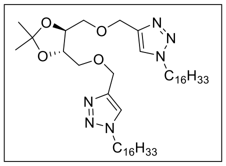

Compound **9a** (500 mg, 2.09 mmol), 1-azidohexadecane (1.12 g, 4.17 mmol), CuSO$_4$ (209 mg, 0.84 mmol), sodium ascorbate (333 mg, 1.67 mmol) were taken in 40 mL stainless steel cup and rest of the procedure was followed as mentioned above. 1.378 g; yield: 85%; colourless solid; 65 ºC; [α]$_D$: -3.9 (*c* 1,CHCl$_3$); ^{1}H NMR (400 MHz, CDCl$_3$) δ 7.57 (1H, s, triazole-**H**), 4.70 (2H, q, J = 12.68 Hz, OC**H**$_2$C$_2$HN$_3$), 4.35 (2H, t, J = 7.24 Hz, C**H**$_2$(CH$_2$)$_{14}$CH$_3$), 3.99-3.98 (1H, m, C**H**OC(CH$_3$)$_2$), 3.67 (2H, dd, J_1 = 3.68 Hz, J_2 = 3.24 Hz, C**H**$_2$CHOC(CH$_3$)$_2$), 1.91 (2H, t, J = 7.08 Hz, CH$_2$C**H**$_2$(CH$_2$)$_{13}$CH$_3$), 1.42 (3H, s, CHOC(C**H**$_3$)$_2$), 1.33-1.26 (26H, bm, CH$_2$(C**H**$_2$)$_{14}$CH$_3$), 0.91 (3H, t, J = 6.68 Hz, CH$_2$(CH$_2$)$_{14}$C**H**$_3$); ^{13}C NMR (100 MHz, CDCl$_3$) δ 144.77, 122.39, 109.78, 77.29, 71.00, 65.06, 50.37, 31.91, 30.31, 29.69, 29.65, 29.60, 29.53, 29.40, 29.35, 29.01, 26.99, 26.52, 22.68, 14.11; IR (Neat) ν_{max} 2919,

2848, 1682, 1464, 1380, 1217, 1153, 1089, 1029, 852, 788, 723; MS LCQ: *m/z* calculated for $C_{45}H_{84}N_6O_4$: [M] 772.66 Found: [M] 772.7.

(viii) (2*S*,3*S*)-2,3-*O*-Isopropylidene-1,4-bis-((1-hexadecyltriazol-4-yl)methoxy)butane (15b)

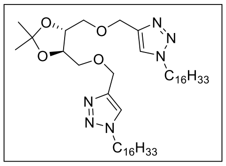

Compound **9b** (600 mg, 2.52 mmol), 1-azidohexadecane (1.34 g, 5.02 mmol), $CuSO_4$ (250 mg, 1.00 mmol), sodium ascorbate (399 mg, 1.51 mmol) were taken in 40 mL stainless steel cup and rest of the procedure was followed as mentioned above. 1.655 g; yield: 85%; colourless solid; 74 ºC; $[\alpha]_D$: 3.9 (*c* 1,$CHCl_3$); ^{1}H NMR (400 MHz, $CDCl_3$) δ 7.57 (1H, s, triazole-**H**), 4.70 (2H, q, J = 12.68 Hz, OC**H**$_2C_2HN_3$), 4.35 (2H, t, J = 7.24 Hz, C**H**$_2(CH_2)_{14}CH_3$), 3.99-3.98 (1H, m, C**H**OC$(CH_3)_2$), 3.67 (2H, dd, J_1 = 3.44 Hz, J_2 = 3.04 Hz, C**H**$_2$CHOC$(CH_3)_2$), 1.91 (2H, t, J = 7 Hz, CH_2C**H**$_2(CH_2)_{13}CH_3$), 1.41 (3H, s, CHOC(C**H**$_3)_2$), 1.33-1.26 (27H, bm, CH_2(C**H**$_2)_{14}CH_3$), 0.89 (3H, t, J = 1.66 Hz, $CH_2(CH_2)_{14}$C**H**$_3$); ^{13}C NMR (100 MHz, $CDCl_3$) δ 144.77, 122.41, 109.80, 77.28, 71.00, 65.06, 50.38, 31.92, 30.32, 29.69, 29.65, 29.61, 29.54, 29.41, 29.36, 29.02, 26.99, 26.52, 22.69, 14.12; MS LTQ: *m/z* calculated for $C_{45}H_{84}N_6O_4$: $[M+Na]^+$ 795.64 Found: $[M+Na]^+$ 795.58.

(ix) (2*R*,3*R*)-2,3-*O*-Isopropylidene-1,4-bis-((1-octadecyltriazol-4-yl)methoxy)butane (16a)

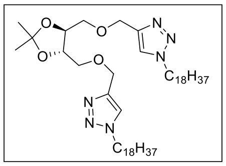

Compound **9a** (500 mg, 2.09 mmol), 1-azidooctadecane (1.23 g, 4.17 mmol), $CuSO_4$ (209 mg, 0.84 mmol), sodium ascorbate (333 mg, 1.67 mmol) were taken in 40 mL stainless steel cup and rest of the procedure was followed as mentioned above. 1.477 g; yield: 85%; colourless solid; 70 ºC; $[\alpha]_D$: -3.0 (*c* 1,$CHCl_3$); ^{1}H NMR (400 MHz, $CDCl_3$) δ 7.57 (1H, s, triazole-**H**), 4.71 (2H, q, J = 7.24 Hz, OC**H**$_2C_2HN_3$), 4.36 (2H, t, J = 7.24 Hz, C**H**$_2(CH_2)_{16}CH_3$), 4.00-3.98 (1H, m, C**H**OC$(CH_3)_2$), 3.67 (2H, t, J_1 = 2.08 Hz, J_2 = 1.36 Hz, C**H**$_2$CHOC$(CH_3)_2$), 1.91 (2H, t, J = 7.04 Hz, CH_2C**H**$_2(CH_2)_{15}CH_3$), 1.43-1.42 (3H, m, CHOC(C**H**$_3)_2$), 1.35-1.27 (30H, bm, CH_2(C**H**$_2)_{16}CH_3$), 0.90 (3H, t, J = 6.64 Hz, $CH_2(CH_2)_{16}$C**H**$_3$); ^{13}C NMR (100 MHz, $CDCl_3$) δ 144.78, 122.40, 109.80, 77.29, 71.01, 65.05, 50.39, 31.92, 30.32, 29.70, 29.65, 29.61, 29.54, 29.41, 29.36, 29.02, 26.99, 26.5, 22.69, 14.11; IR (Neat) ν_{max} 3130, 2919, 2848, 1682, 1463, 1371, 1337, 1217, 1152, 1088, 1059, 852, 788, 724; MS LTQ: *m/z* calculated for $C_{49}H_{92}N_6O_4$: $[M+Na]^+$ 851.71 Found: $[M+Na]^+$ 851.71.

(x) (2*S*,3*S*)-2,3-*O*-Isopropylidene-1,4-bis-((1-octadecyltriazol-4-yl)methoxy)butane (16b)

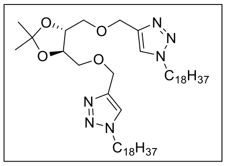

Compound **9b** (600 mg, 2.09 mmol), 1-azidooctadecane (1.24 g, 4.17 mmol), $CuSO_4$ (209 mg, 0.84 mmol), sodium ascorbate (333 mg, 1.67 mmol) were taken in 40 mL stainless steel cup and rest of the procedure was followed as mentioned above. 1.566 g; yield: 90%; Colourless solid; 78 ºC; $[\alpha]_D$: 2.3 (*c* 1,$CHCl_3$); ^{1}H NMR (400 MHz, $CDCl_3$) δ 7.57 (1H, s, triazole-H), 4.71 (2H, q, *J*= 7.24, OC**H**$_2$C$_2$HN$_3$), 4.36 (2H, t, *J* = 7.24 Hz, C**H**$_2$(CH$_2$)$_{16}$CH$_3$), 4.00-3.98 (1H, m, C**H**OC(CH$_3$)$_2$), 3.67 (2H, dd, J_1 = 3.28 Hz, J_2 = 3.04 Hz, C**H**$_2$CHOC(CH$_3$)$_2$), 1.91 (2H, t, *J* = 6.96 Hz, CH$_2$C**H**$_2$(CH$_2$)$_{15}$CH$_3$), 1.42 (3H, m, CHOC(C**H**$_3$)$_2$), 1.33-1.26 (31H, bm, CH$_2$(C**H**$_2$)$_{16}$CH$_3$), 0.90 (3H, t, *J* = 6.64 Hz, CH$_2$(CH$_2$)$_{16}$C**H**$_3$); ^{13}C NMR (100 MHz, $CDCl_3$) δ 144.76, 122.42, 109.81, 77.27, 71.00, 65.06, 50.39, 31.93, 30.33, 29.71, 29.67, 29.62, 29.55, 29.42, 29.37, 29.03, 27.00, 26.53, 22.70, 14.14; MS LTQ: *m/z* calculated for $C_{49}H_{92}N_6O_4$: $[M+Na]^+$ 851.71 Found: $[M+Na]^+$ 851.51.

3. Gelation Study

Gelation was performed by dissolving 0.7 mg (*R, R* -derivatives) and 1.5 mg (*S, S* -derivatives) in 1 ml of HPLC grade solvents viz. *n*-hexane, *n*-heptane, *n*-octane, methanol, ethanol dichloromethane, toluene, DMSO, water and 1:1 mixtures of dichloromethane/*n*-hexane, *n*-hexane/water and methanol/water in a 2 ml glass vials with heating. After complete dissolution, solutions were allowed to remain undisturbed at room temperature for 10 min to obtain gels.

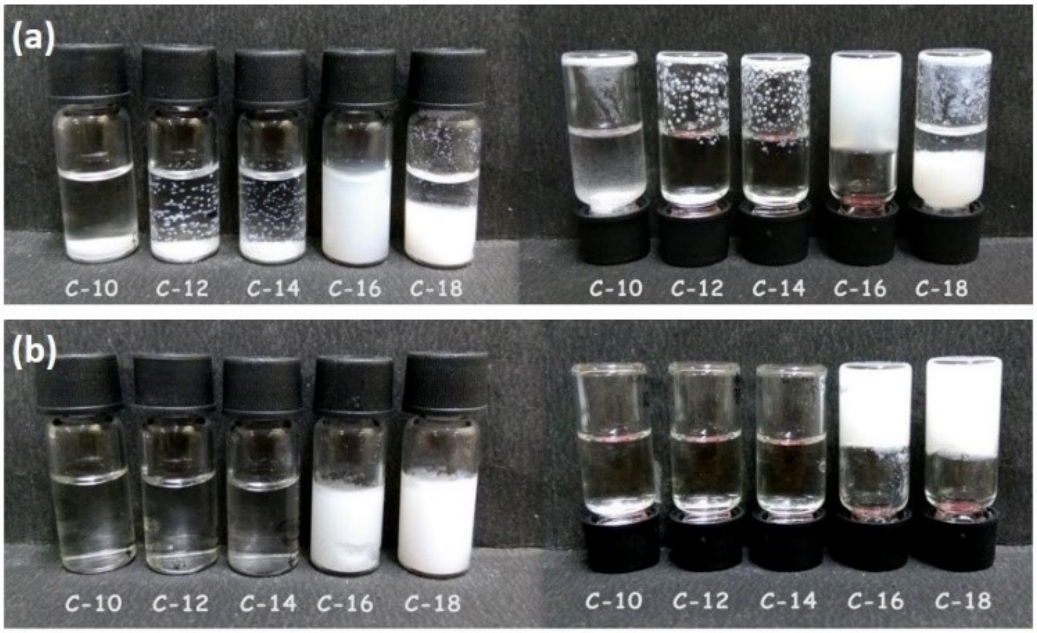

Fig. S1. Gelation of ***R, R*** -tetritol based triazole linked lipid derivatives in (a) *n*-heptane and (b) methanol.

6. Microscopic study

To get clear TEM images of nanostructures and to avoid occlusion of copper grids by densely packed gel fibres the gel sample was diluted to 0.02% *w/v*. Then, 5 µL aliquot of the suspension of n-hexane/n-heptane was drop casted on a clean copper grid, the solvent was evaporated completely and the sample was examined under TEM. In case of solvents which does not result organogels such as dicholoromethane and toluene, the samples were not diluted, instead 0.7 %*w/v* concentration was used and directly placed on copper grid, dried and observed under TECNAI G^2 F-20 high resolution transmission electron microscope (HR-TEM) from FEI.

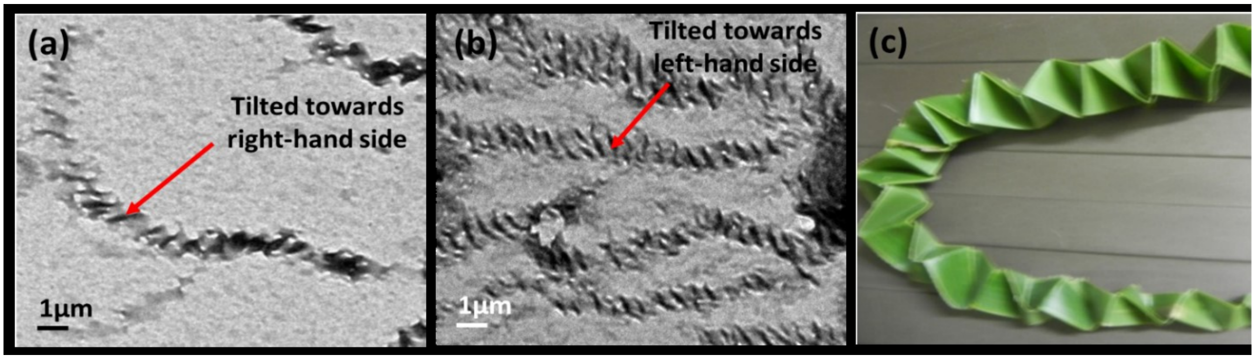

Fig. S2. TEM images of self-assembly formed by (a) **15a** and (b) **15b** in CH_2Cl_2 (0.02 %w/v); (c) Photograph of a traditional palm leaf-based origami-type craft-work done in coconut palm leaf shown for comparison.

7. Circular dichroism spectra

Circular dichroism spectra were recorded using a quartz cell of 1 mm pathlength and data pitch 0.1 nm with a scanning speed of 50 nm/min. For the CD studies of the gels, a weak gel with a concentration of 1.5 mM was first prepared and CD spectra were recorded at 25 ºC. Each spectrum is the average of two consecutive scans. The spectra show mirror images of the cotton band for the diluted gels of **15a**, **15b** in methanol, suggesting supramolecular chiral nature of the assembly.

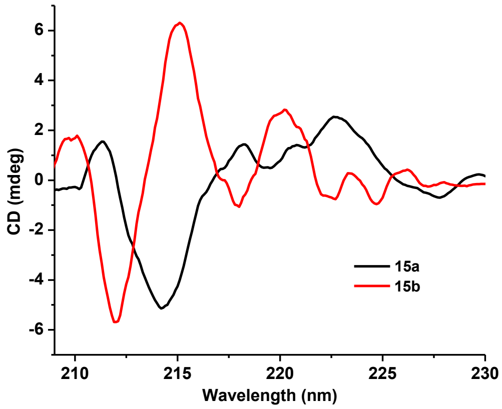

Fig. S3. CD spectra of the diluted gel from (a) **15a** and (b) **15b** in methanol (1.5 mM) suggesting presence of supramolcular chirality.

8. XRD Analysis

The organogels in *n*-heptane were heated to sol, and 100 μL of the sols were individually transferred carefully on a pre-cleaned glass slide and left to air dry for 8 h to form the self-supported cast films on which measurements were performed using a Model-D8 Advance X-ray diffractometer. The X-ray beam generated with a Cu anode at the wavelength of $K_{\alpha 1}$ beam at 1.5418 Å was directed toward the film edge, and scanning was done upto a 2θ value of 22°. Data were analysed and interpreted in terms of higher order reflections.

The low intensity peaks at higher angles indicate higher order of packing in the arrangement of gel networks.

Table S1. XRD parameter of the gels of **15a** in different solvents and with metal ion

	2θ values (degree)	d spacing (nm)
**Gel in n-hexane (15a)**	2.97	2.98
	5.99	1.47
	11.92	0.74
	14.84	0.59
**Gel in methanol (15a)**	2.97	2.98
	4.43	1.99
	5.99	1.47
	11.92	0.74
	13.34	0.66
	14.84	0.59
**Gel in methanol (15a) + Cu^{2+}**	2.53	2.48
	7.49	0.83
	10.02	0.62
	12.90	0.48

9. Rheological studies

Rheological studies were performed on an Anton Paar MCR 302 rheometer with an adjustable peltier temperature controlling system using a cone and plate geometry of CP-50-1. The gap distance between the cone and the plate was fixed at 0.1 mm. Oscillatory frequency sweep experiments were performed at constant amplitude of 0.05% strain which corresponds to the linear viscoelastic region of gel samples for an angular frequency range 200 to 0.001 rad/s at 20 ºC. Stress amplitude sweep experiments were performed at a constant oscillation frequency of 10 rad/s for the strain range 0.001 to 100 at 20 ºC. Temperature ramp measurements were performed at a

constant oscillation frequency of 10 rad/s and constant oscillation amplitude of 0.05% strain for a temperature range of 20 to 50 °C at a heating and cooling rate of 5 °C/min.

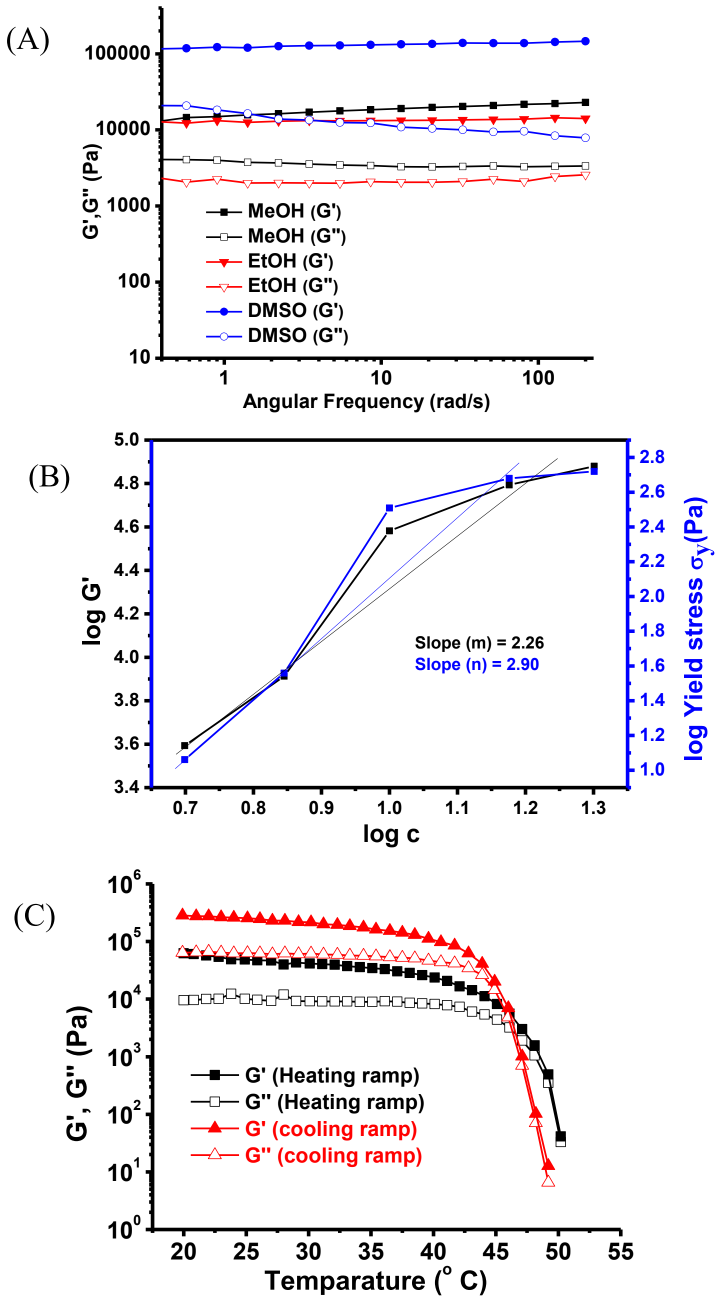

Fig. S4. (A) Comparative oscillatory amplitude sweep measurement for the gel made in methanol, ethanol and DMSO with **15a** (1 %w/v). (B) A typical plot of logG' vs logC and logσ_y *vs.* logC to show the power law behavior for the gels in methanol. (C) Typical temperature ramp measurements for gel made in methanol at a constant angular frequency of 10 rad/s and constant amplitude of 0.05% strain for a temperature range of 20 to 50 °C at a heating and cooling rate of 5 °C/min.

10. Drug Entrapment study:

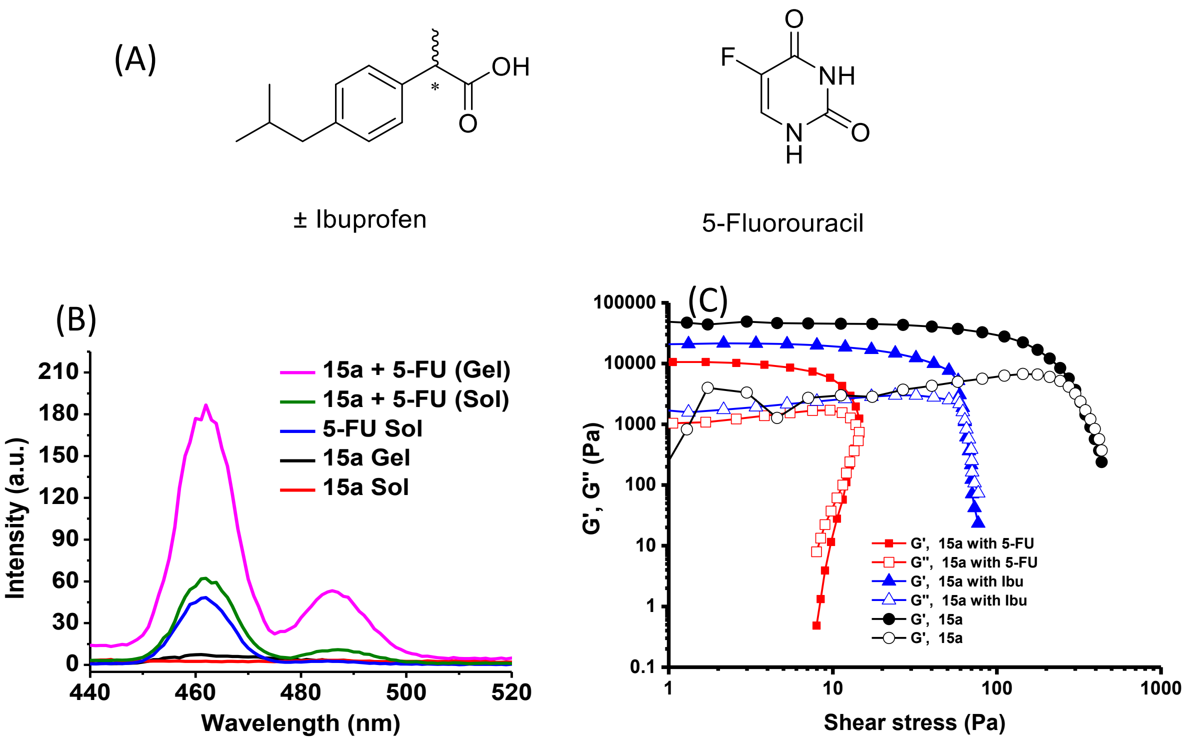

Figure S5. (A) Chemical structure of ibuprofen and 5-fluorouracil. (B) Fluorescence emission spectra of 5-Fluorouracil in both gel and sol state. (C) Typical oscillatory amplitude sweep for the methanol gels of **15a** (1 %w/v) without drug and with of 5-FU (0.36 %w/v) and Ibu (0.45 %w/v) drugs.

11. In-vitro drug release study

HPLC analysis:

For Ibuprofen:

The samples for calibration curve were prepared by making 10 ml of 1mg/ml stock solution of ibuprofen. 6 dilutions of 100 ppm, 200 ppm, 400 ppm, 600 ppm, 800 ppm and 1000 ppm were prepared and calibration curve as obtained. The samples at different time points were taken and given directly, without dilution, for HPLC analysis. The HPLC was carried out on LC-10AT VP Shimadzu, using Phenomenex®, Luna C18(2) HPLC column (with particle size 5 µ and pore size100 Å). Acetonitrile (A) and phosphoric acid (B) (pH 2.50) in the ratio of A:B = 55:45 were used as mobile phase and the flow rate was kept at 2 ml/min. UV-visible detector SPD-10A VP Shimadzu, was used with the detection wavelength of 230 nm at room temperature. The retention time of ibuprofen was found out to be 7.56 min.

For 5-fluorouracil

The samples for calibration curve were prepared by making 10 ml of 1mg/ml stock solution of 5-fluorouracil. 6 dilutions of 100 ppm, 200 ppm, 400 ppm, 600 ppm, 800 ppm and 1000 ppm were prepared and calibration curve as obtained. The samples at different time points were taken and given directly, without dilution, for HPLC analysis. The HPLC was carried out on LC-10AT VP Shimadzu, using Phenomenex®, Luna C18(2) HPLC column (with particle size 5 μ and pore size100 Å). Acetonitrile (A) and phosphoric acid (B) (pH 2.50) in the ratio of A:B = 55:45 were used as mobile phase and the flow rate was kept at 1 ml/min. UV-visible detector SPD-10A VP Shimadzu, was used with the detection wavelength of 260 nm at room temperature. The retention time of 5-fluorouracil was found out to be 3.24 min. From the drug release profile of ibuprofen and 5-fluorouracil (Fig S6), we observed that the maximum cumulative release of 5-fluorouracil i.e. up to 95% occurred within the first 8 h of the study when water was used as the dissolution media. In the case of ibuprofen the release was relatively slower, and after 36 h only 62% of the drug was found released.

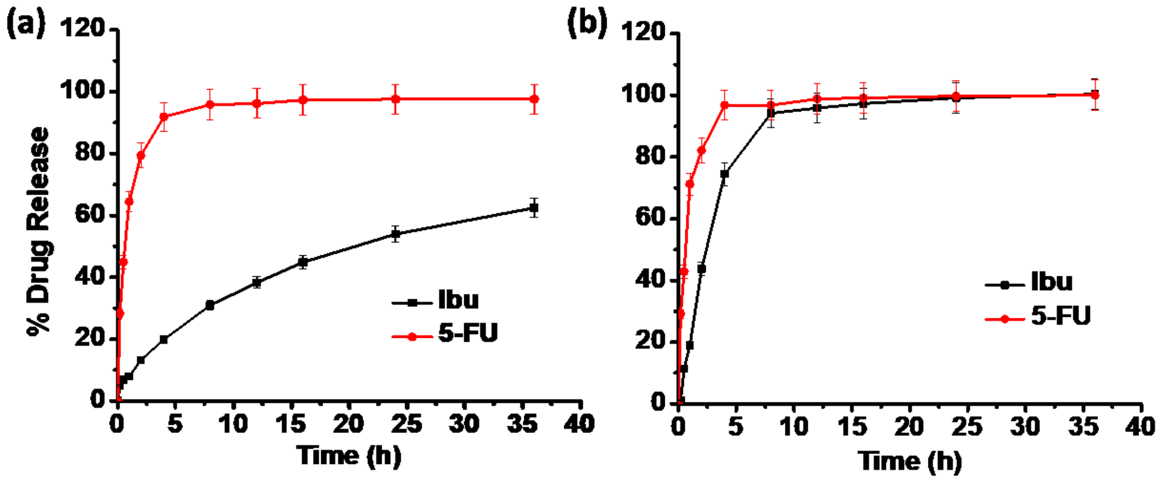

Figure S6. Drug release profile of ibuprofen and 5-fluorouracil in (a) water and (b) phosphate buffer of pH = 7.4.

12. References

1. Kartha, K. P. R. *Tetrahedron Lett.* **1986**, *27*, 3415–3416.
2. Kenso, S.; Hidekazu, O.; Masako, T.; Atushiro, O. *Bull. Chem. Soc. Jpn.* **1984**, *57*, 1948–1953.
3. Brandsma, L. *Preparative acetylinic chemistry*, 2nd ed.; Elsevier, **1988**
4. Zhu, G.; Dordick, J. S., *Chem. Mater.* **2006,** 18, (25), 5988-5995.

^{1}H and ^{13}C NMR spectra of (*2R, 3R*)-diethyl-2,3-*O*-isopropylidene-tartrate **(7a)**

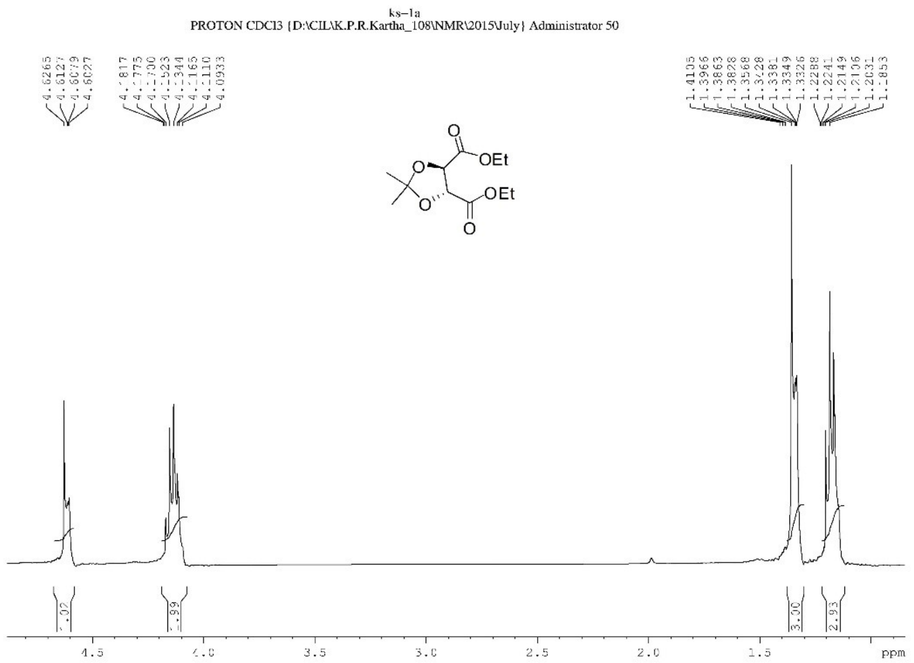

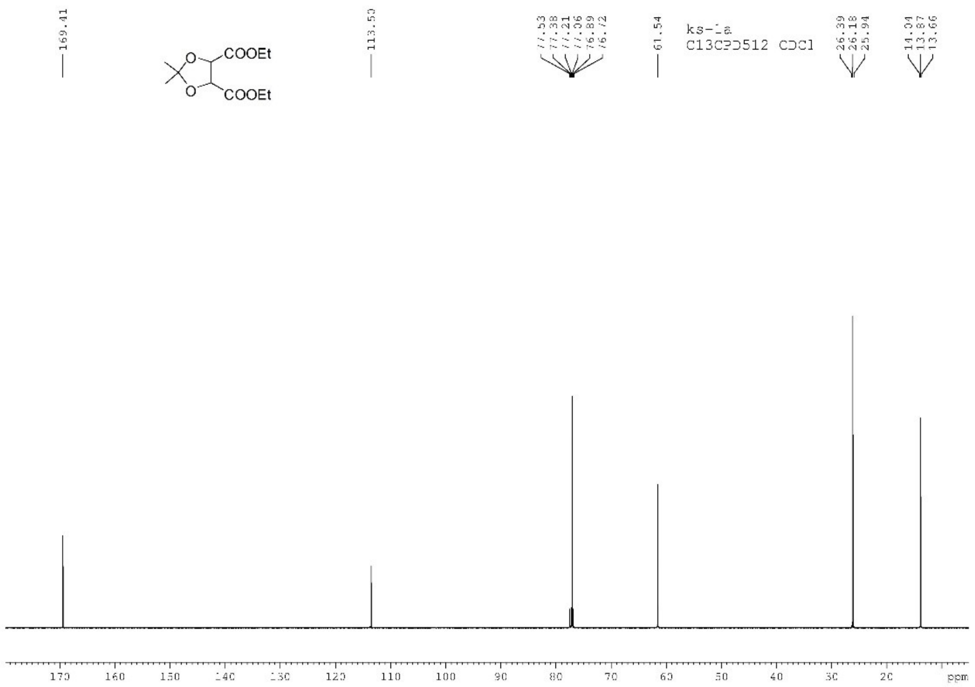

^{1}H and ^{13}C NMR spectra of (*2S, 3S*)-diethyl-2,3-*O*-isopropylidene-tartrate **(7b)**

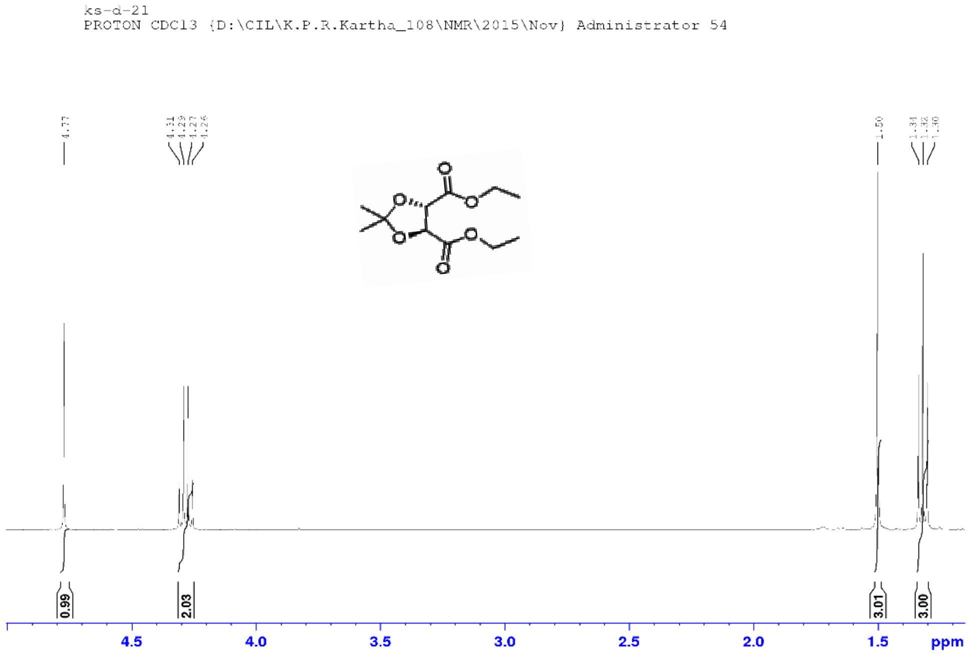

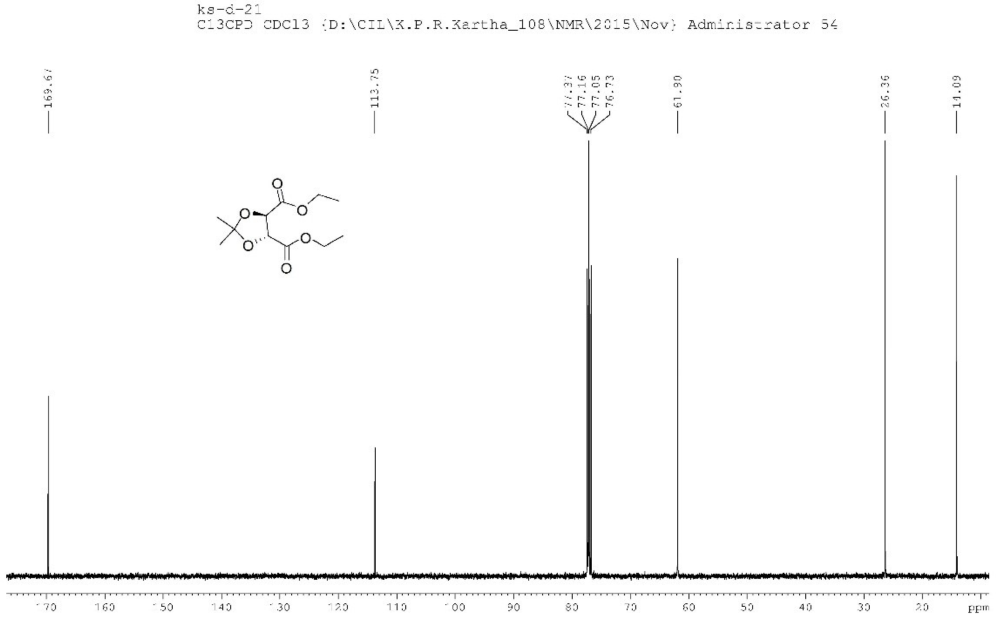

^{1}H and ^{13}C NMR spectra of (*2R, 3R*)-2,3-*O*-isopropylidene-tetritol **(8a)**

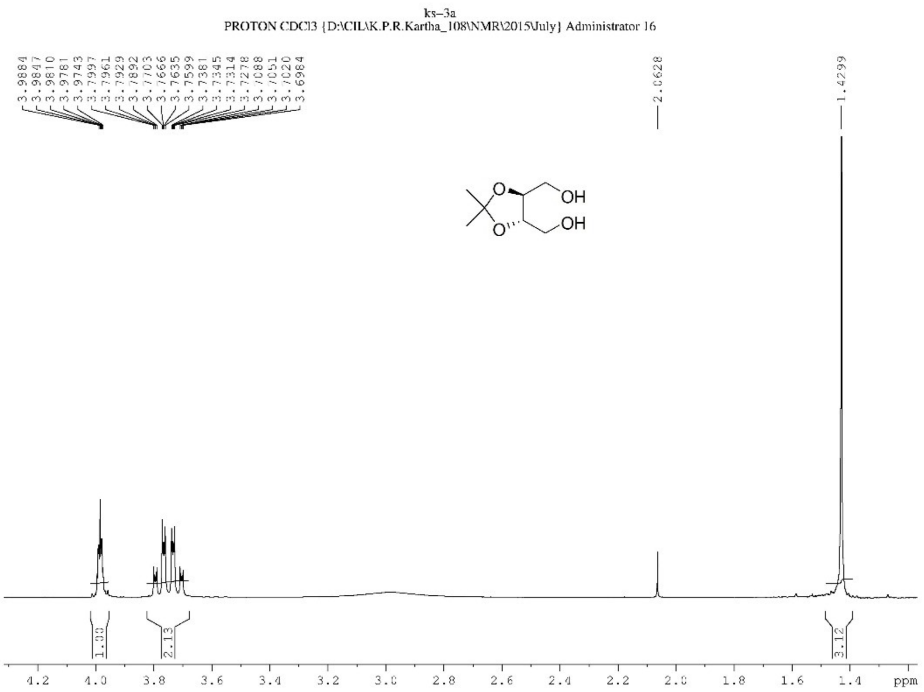

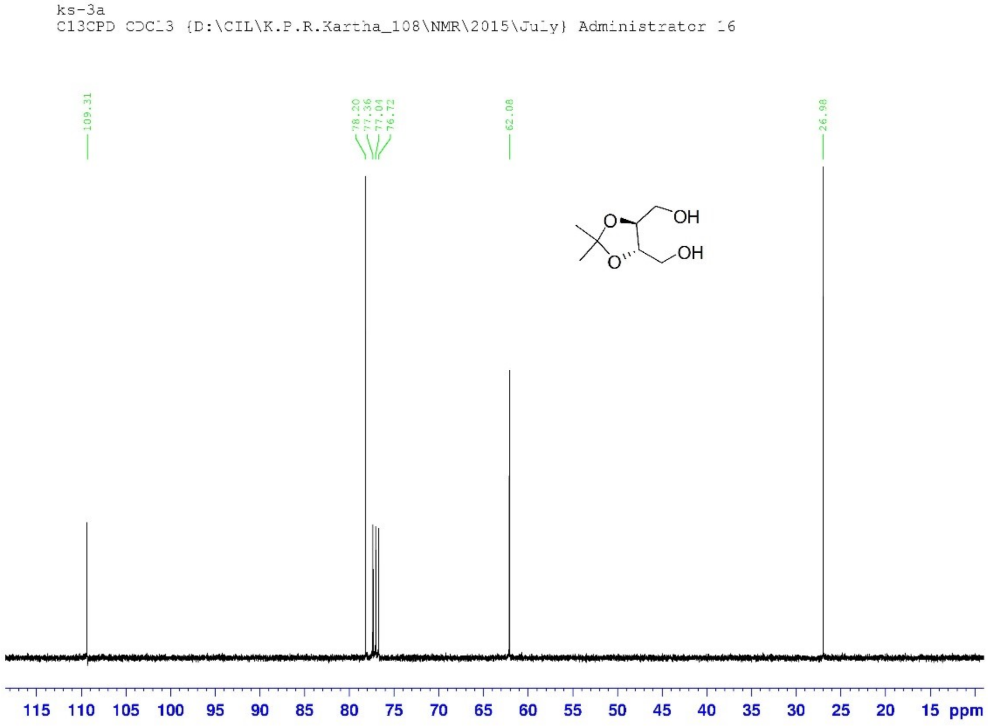

^{1}H and ^{13}C NMR spectra of (*2S, 3S*)-2,3-*O*-isopropylidene-tetritol **(8b)**

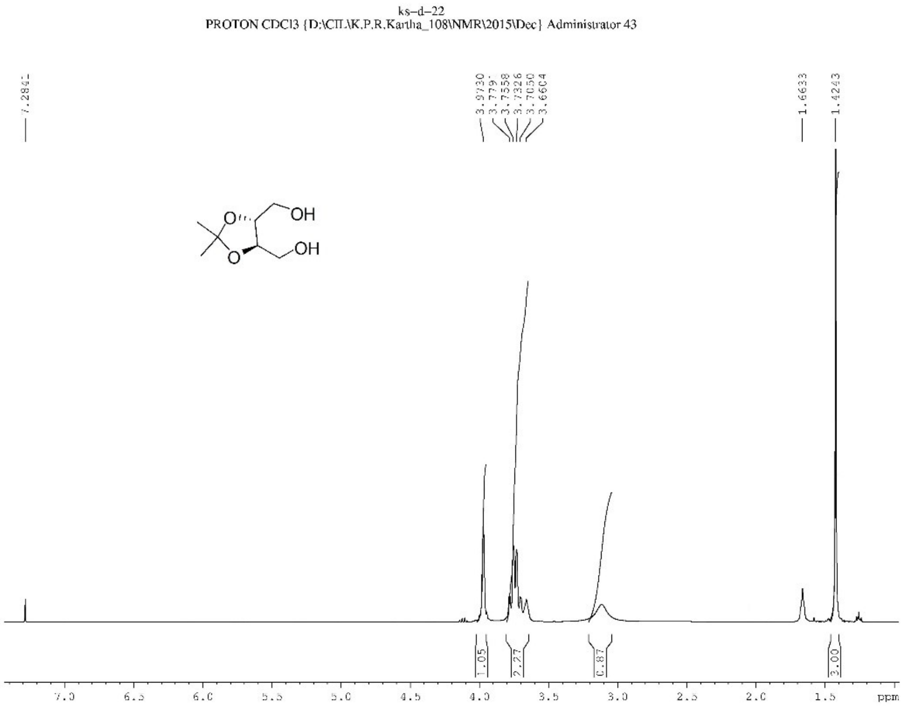

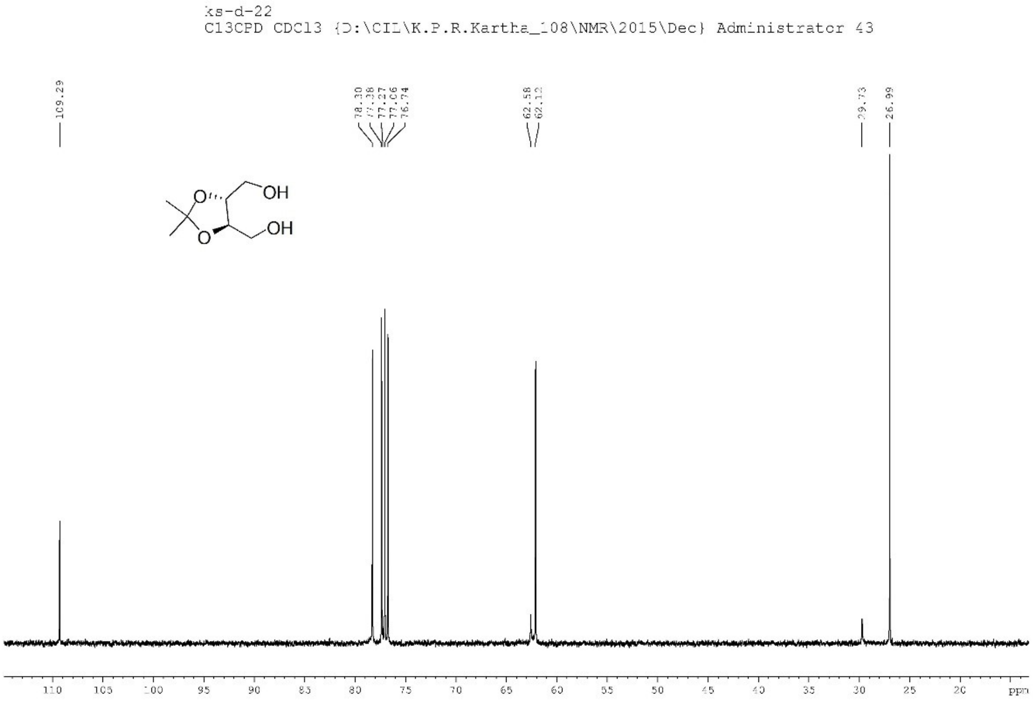

^{1}H and ^{13}C NMR spectra of (*2R, 3R*)-2,3-*O*-isopropylidene-1,4-*O*-dipropargyl-tetritol **(9a)**

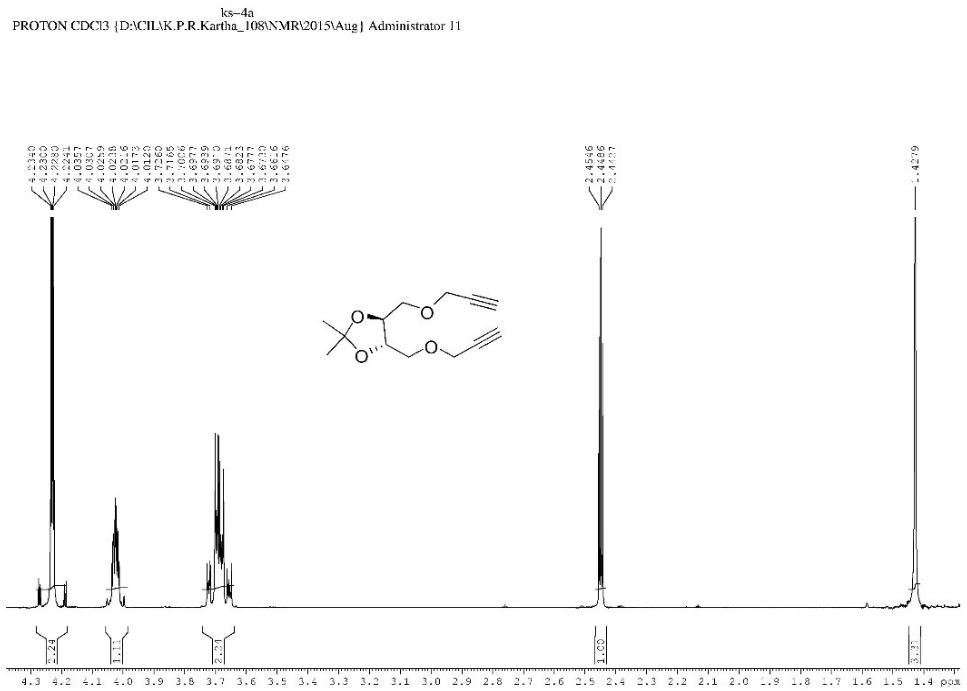

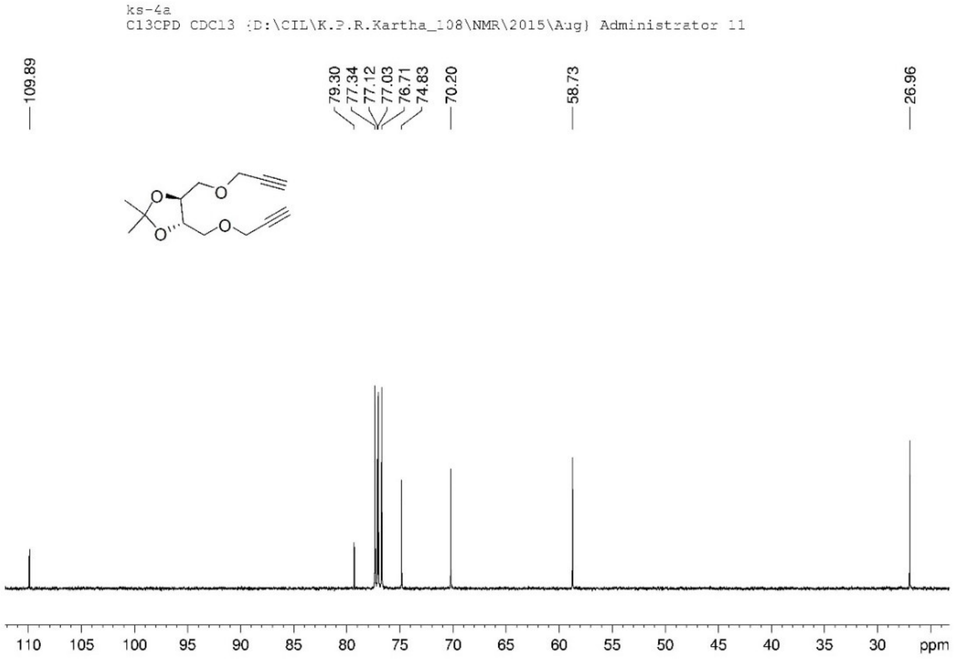

^1H and ^{13}C NMR spectra of (*2S, 3S*)-2,3-*O*-isopropylidene-1,4-*O*-dipropargyl-tetritol **(9b)**

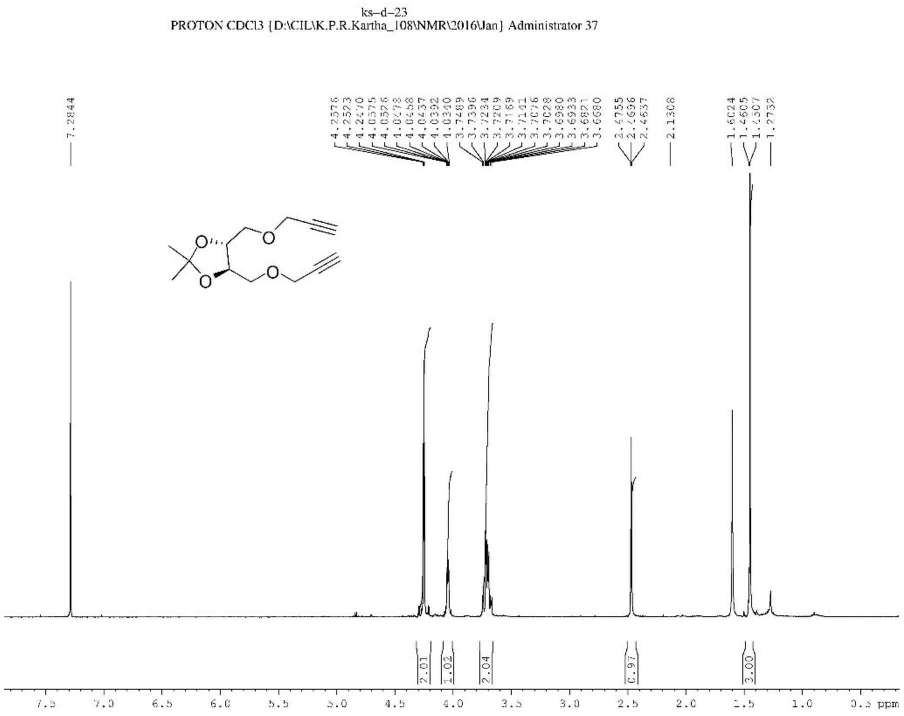

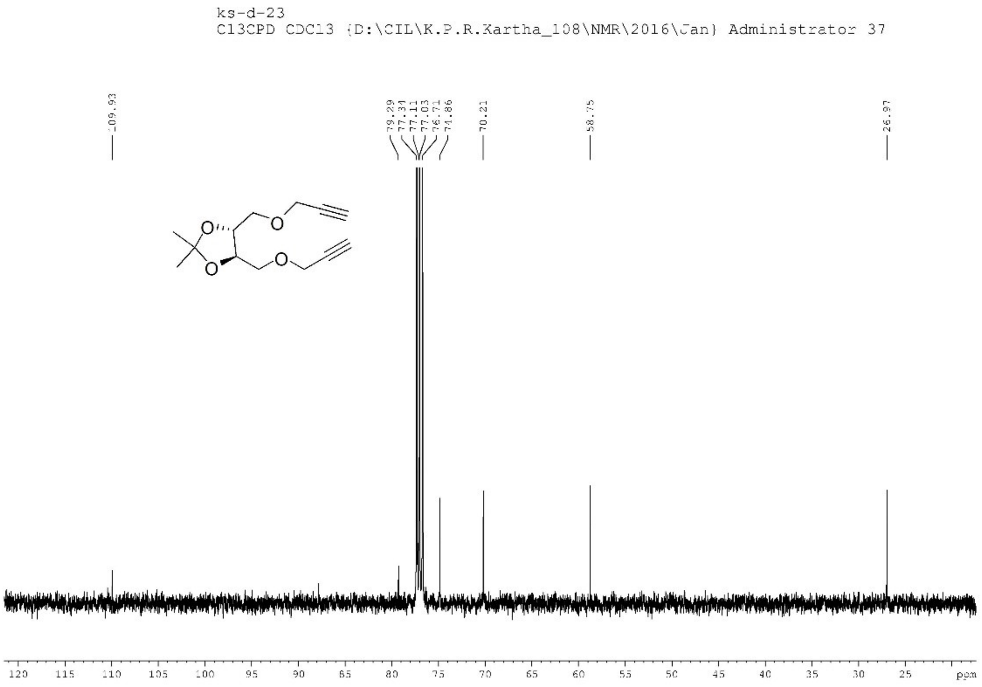

^{1}H and ^{13}C NMR spectra of (2*R*,3*R*)-2,3-*O*-isopropylidene-1,4-bis-((1-decyltriazol-4-yl)methoxy) butane **(12a)**

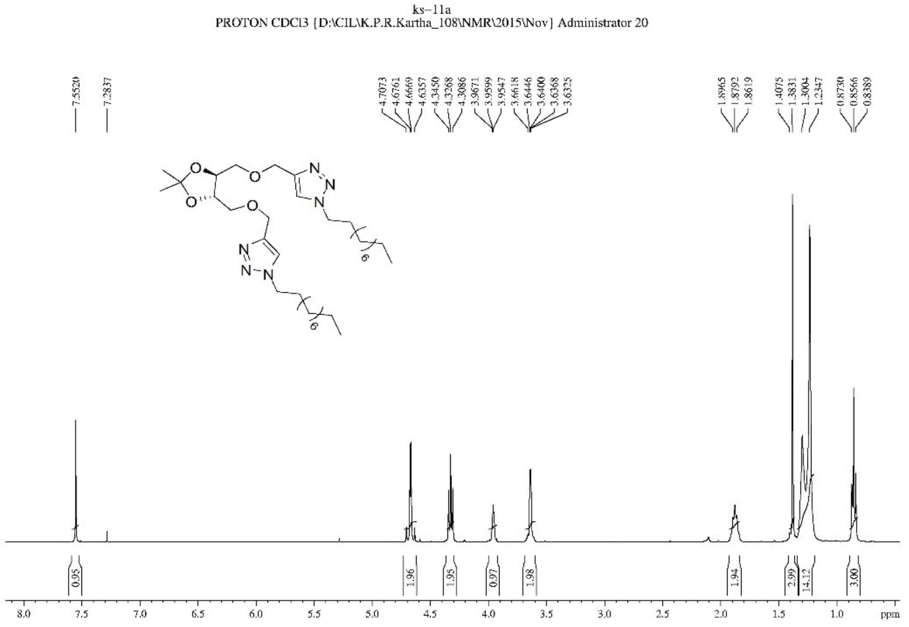

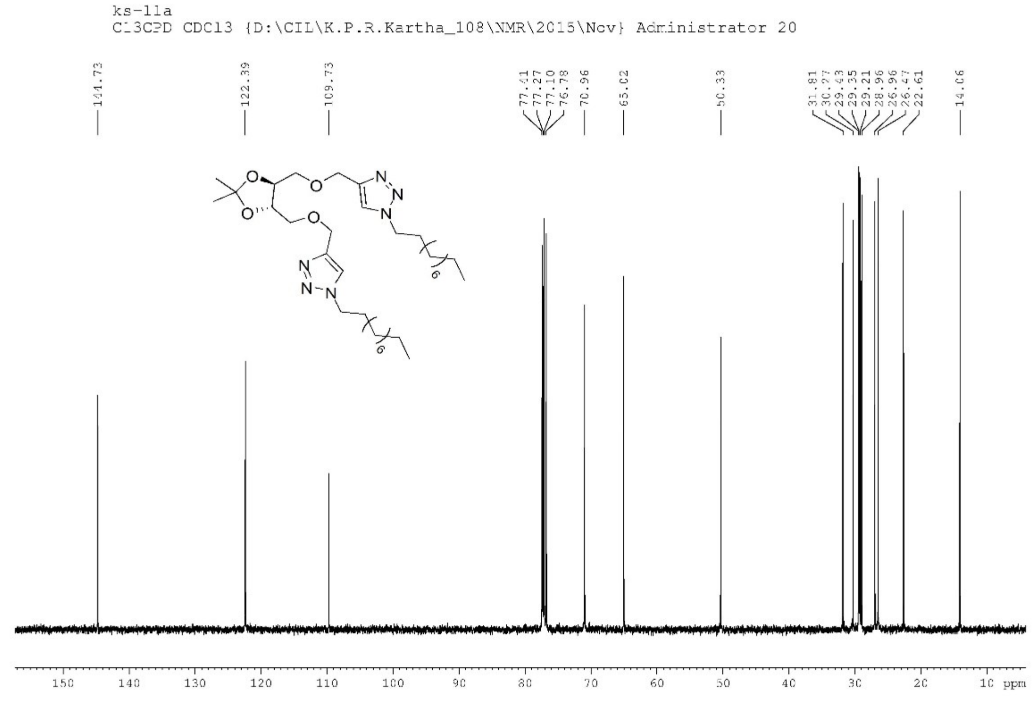

^{1}H and ^{13}C NMR spectra of (2*S*,3*S*)-2,3-*O*-isopropylidene-1,4-bis-((1-decyltriazol-4-yl)methoxy) butane **(12b)**

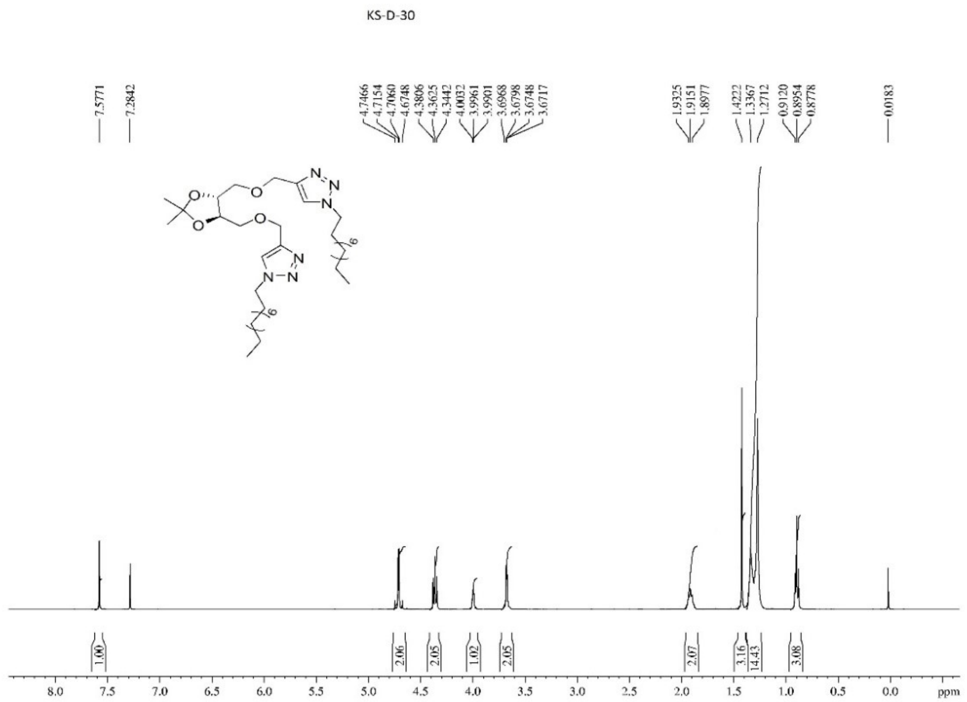

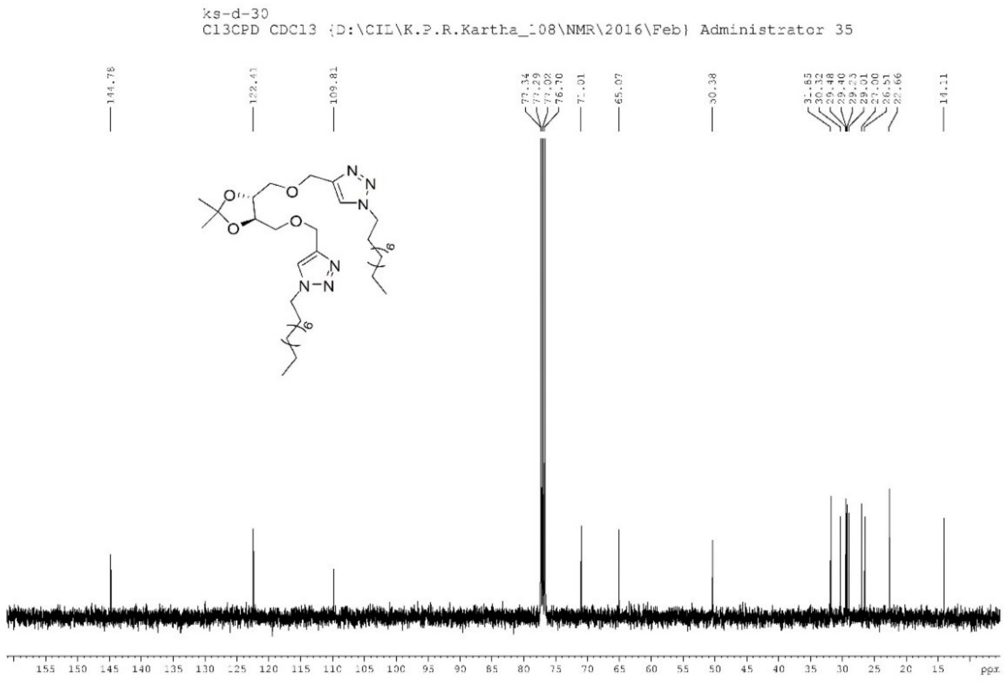

^{1}H and ^{13}C NMR spectra of (2*R*,3*R*)-2,3-*O*-isopropylidene-1,4-bis-((1-dodecyltriazol-4-yl)methoxy) butane **(13a)**

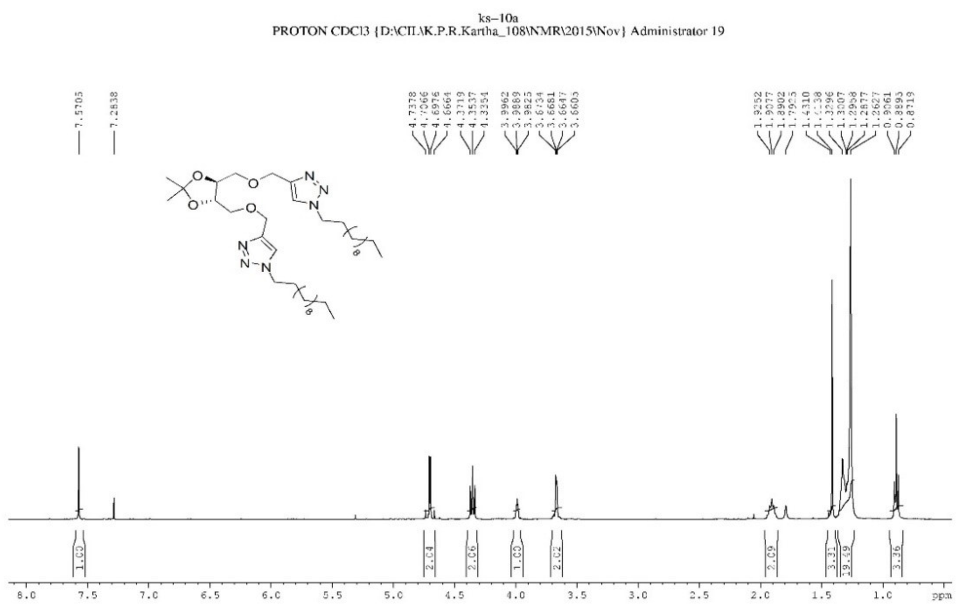

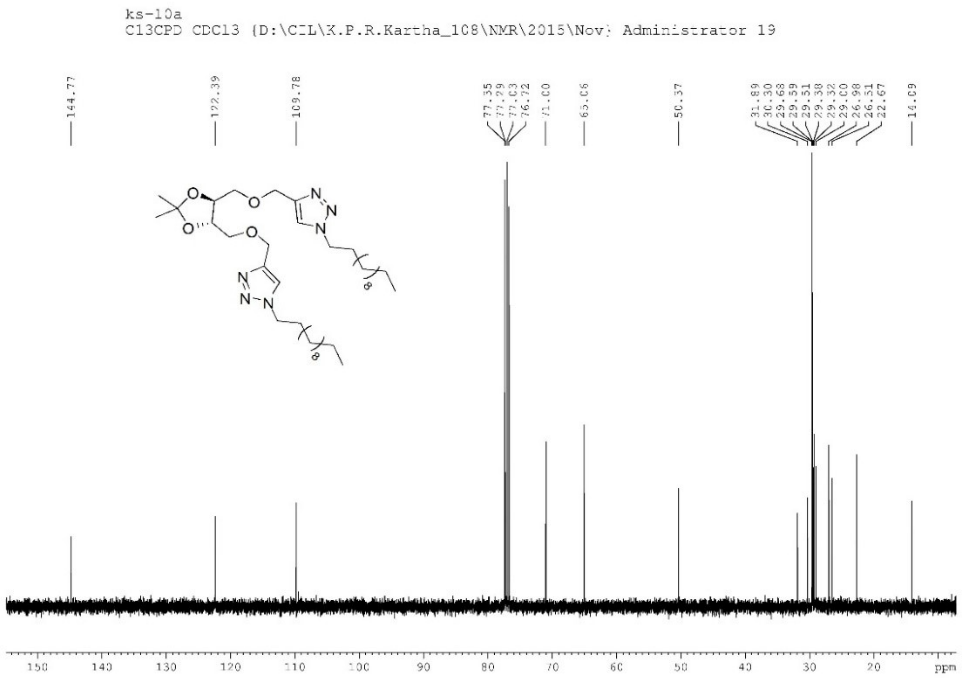

^{1}H and ^{13}C NMR spectra of (2*S*,3*S*)-2,3-*O*-isopropylidene-1,4-bis-((1-dodecyltriazol-4-yl)methoxy) butane **(13b)**

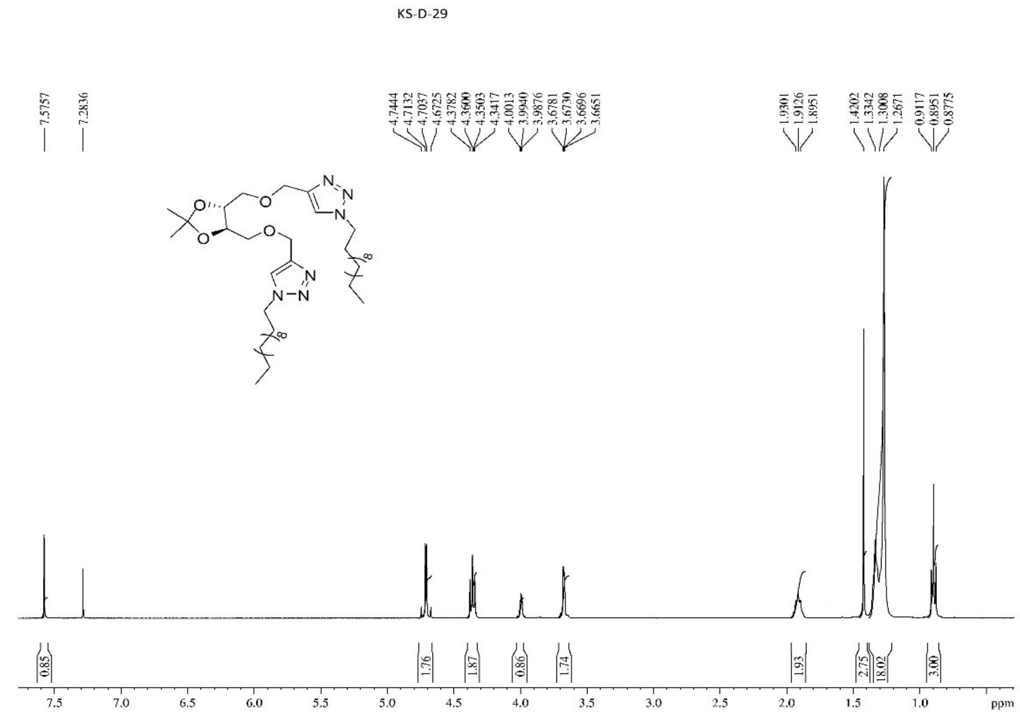

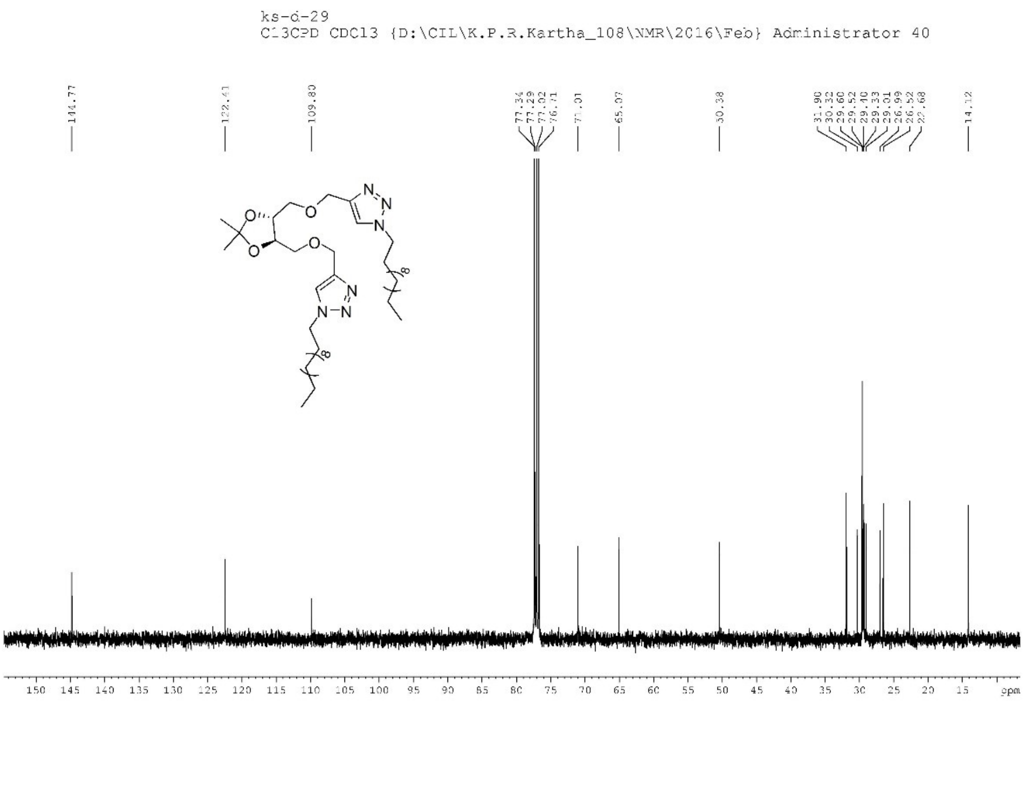

^{1}H and ^{13}C NMR spectra of (2*R*,3*R*)-2,3-*O*-isopropylidene-1,4-bis-((1-tetradecyltriazol-4-yl)methoxy) butane **(14a)**

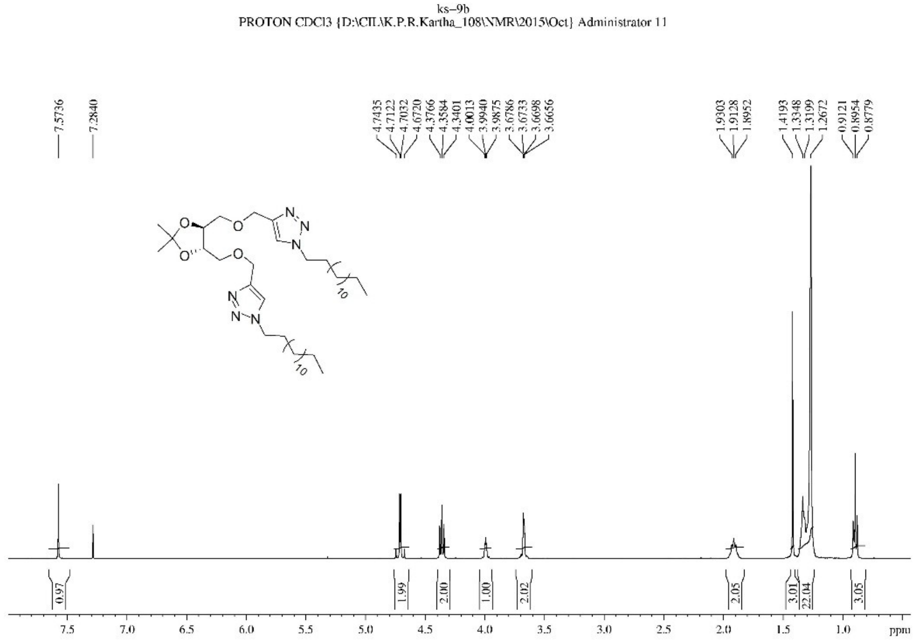

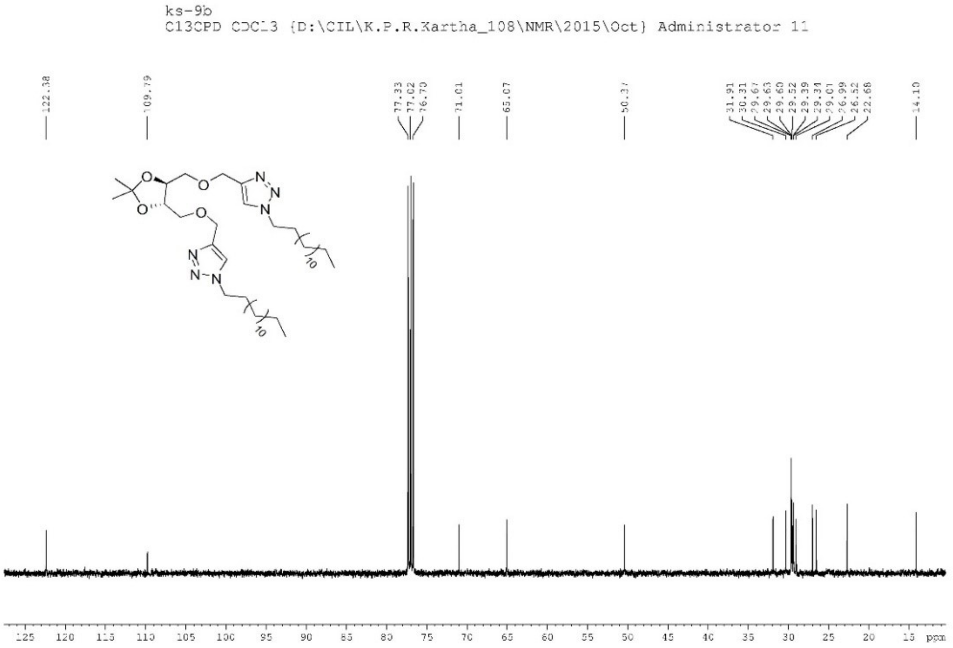

^{1}H and ^{13}C NMR spectra of (2*S*,3*S*)-2,3-*O*-isopropylidene-1,4-bis-((1-tetradecyltriazol-4-yl)methoxy) butane **(14b)**

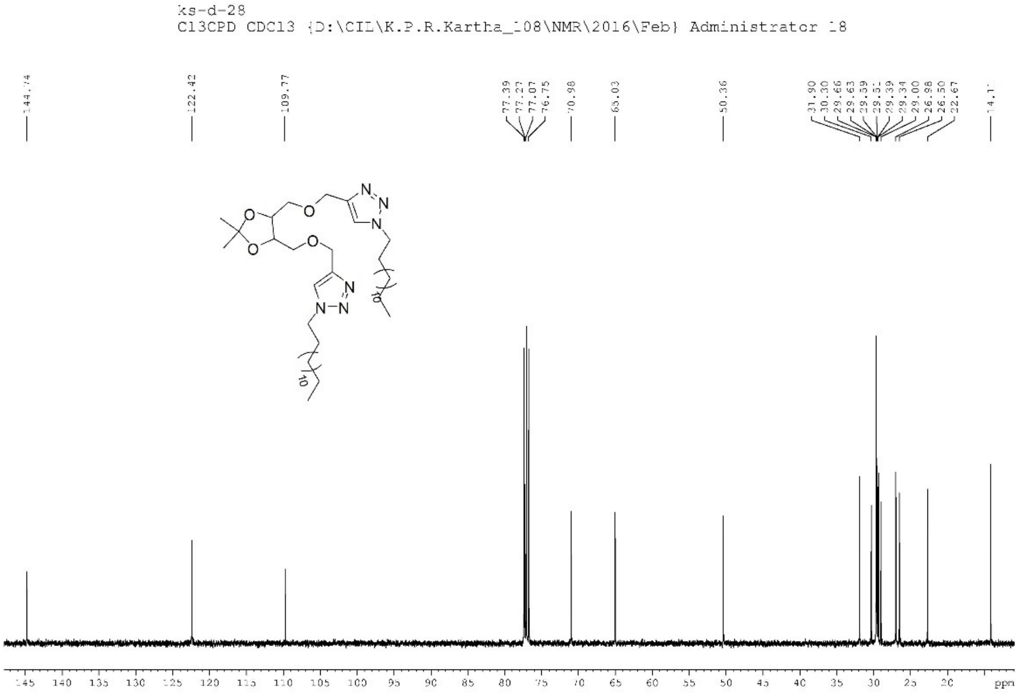

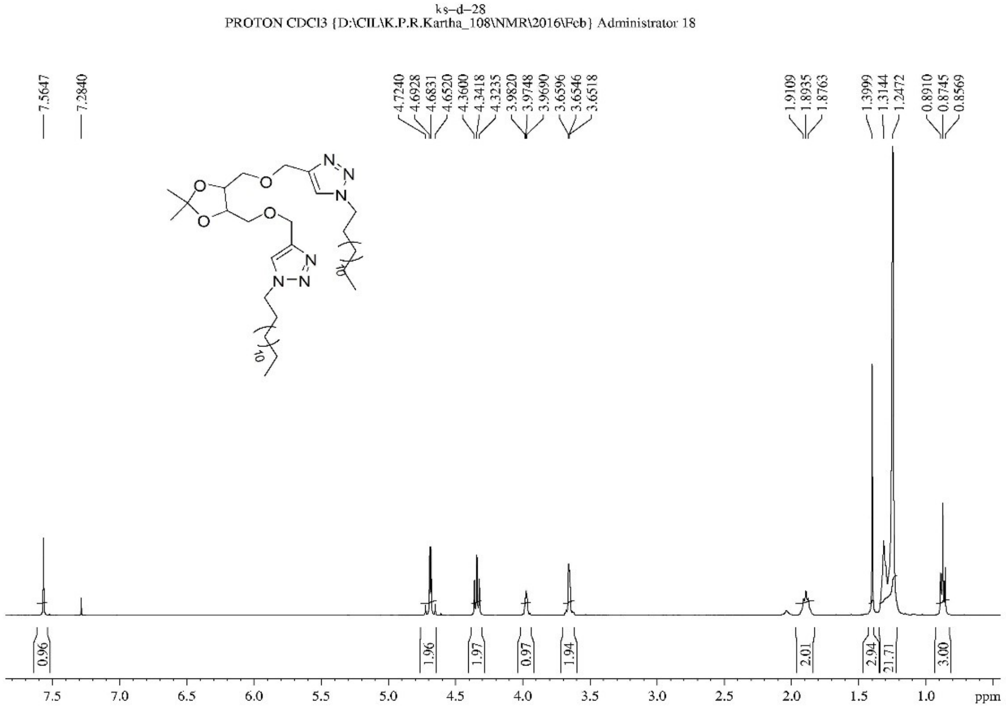

^{1}H and ^{13}C NMR spectra of (2*R*,3*R*)-2,3-*O*-isopropylidene-1,4-bis-((1-hexadecyltriazol-4-yl)methoxy)butane **(15a)**

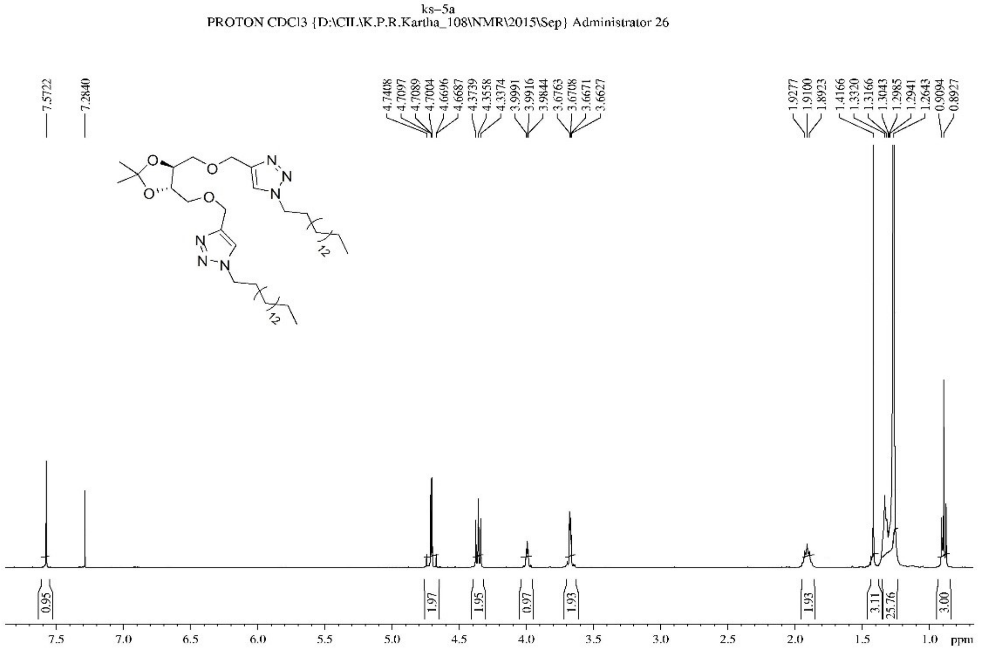

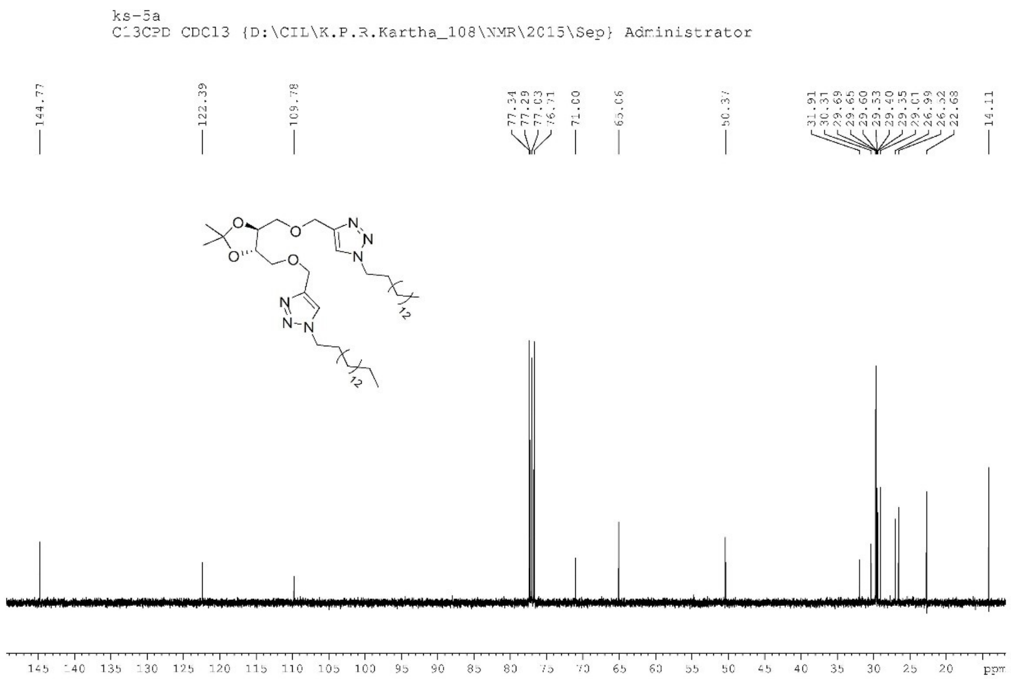

^{1}H and ^{13}C NMR spectra of (2*S*,3*S*)-2,3-*O*-isopropylidene-1,4-bis-((1-hexadecyltriazol-4-yl)methoxy)butane **(15b)**

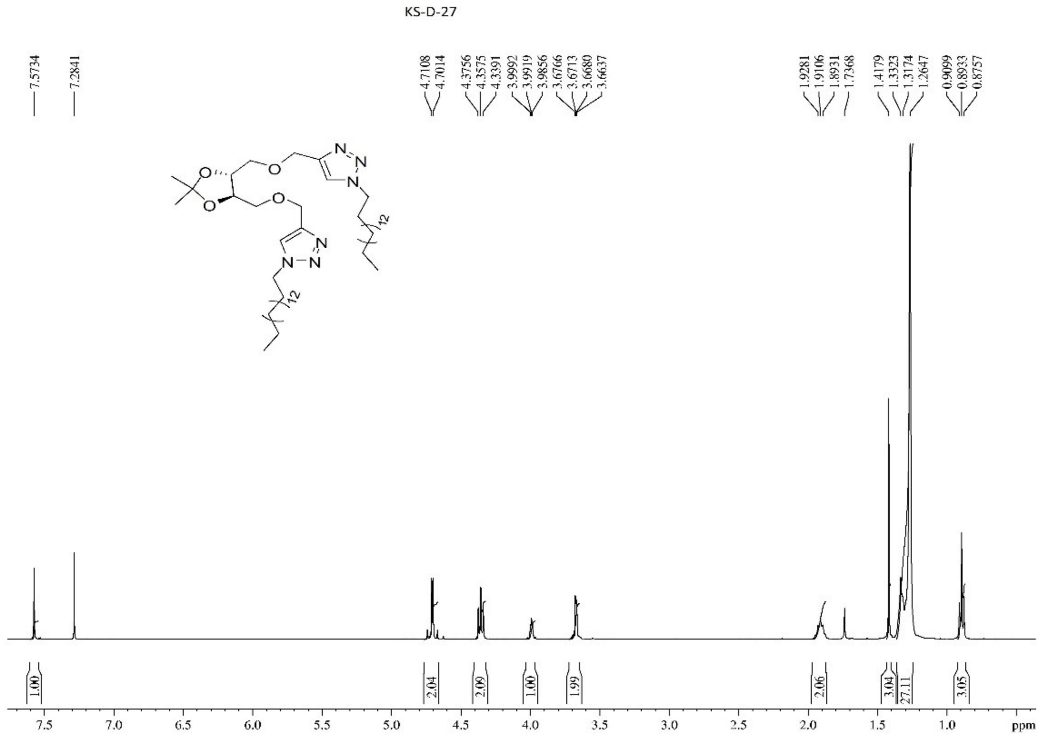

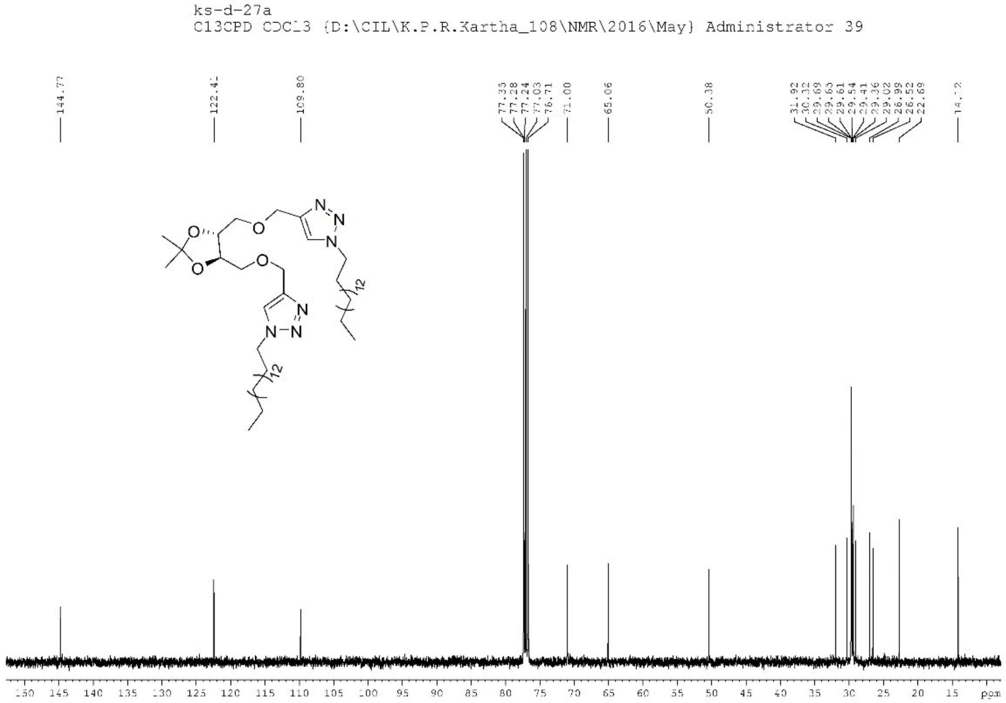

^{1}H and ^{13}C NMR spectra of (2*R*,3*R*)-2,3-*O*-isopropylidene-1,4-bis-((1-octadecyltriazol-4-yl)methoxy)butane **(16a)**

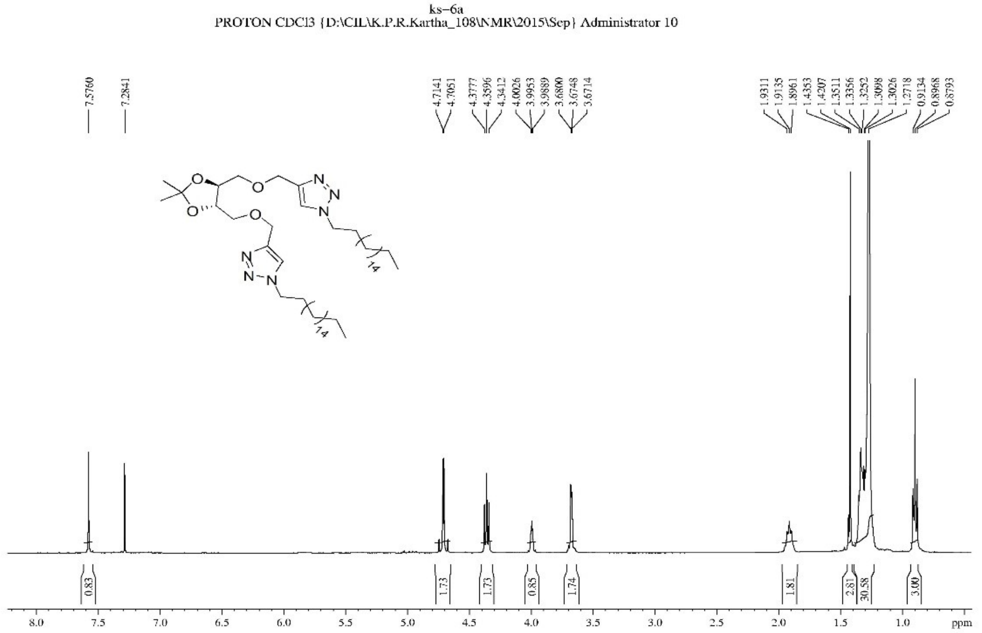

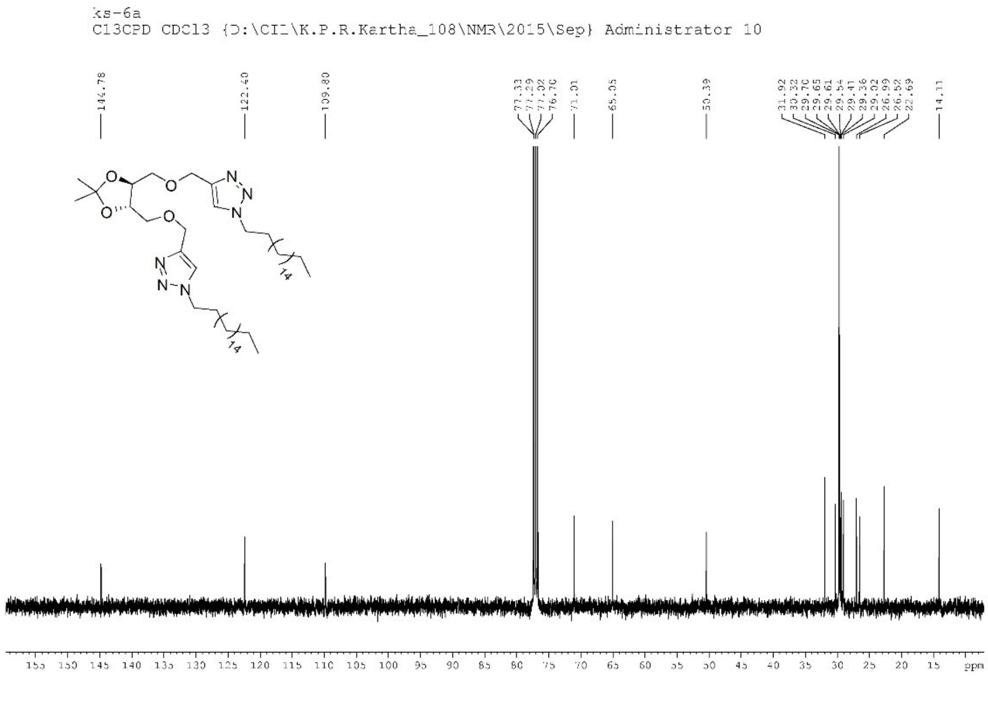

^{1}H and ^{13}C NMR spectra of (2*S*,3*S*)-2,3-*O*-isopropylidene-1,4-bis-((1-octadecyltriazol-4-yl)methoxy)butane **(16b)**

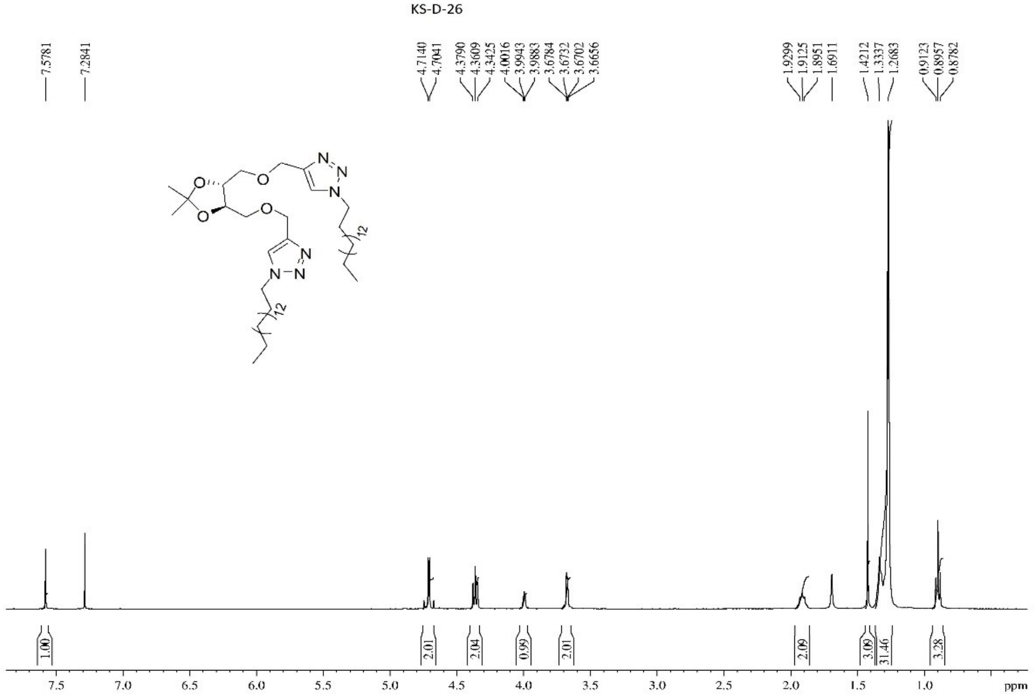

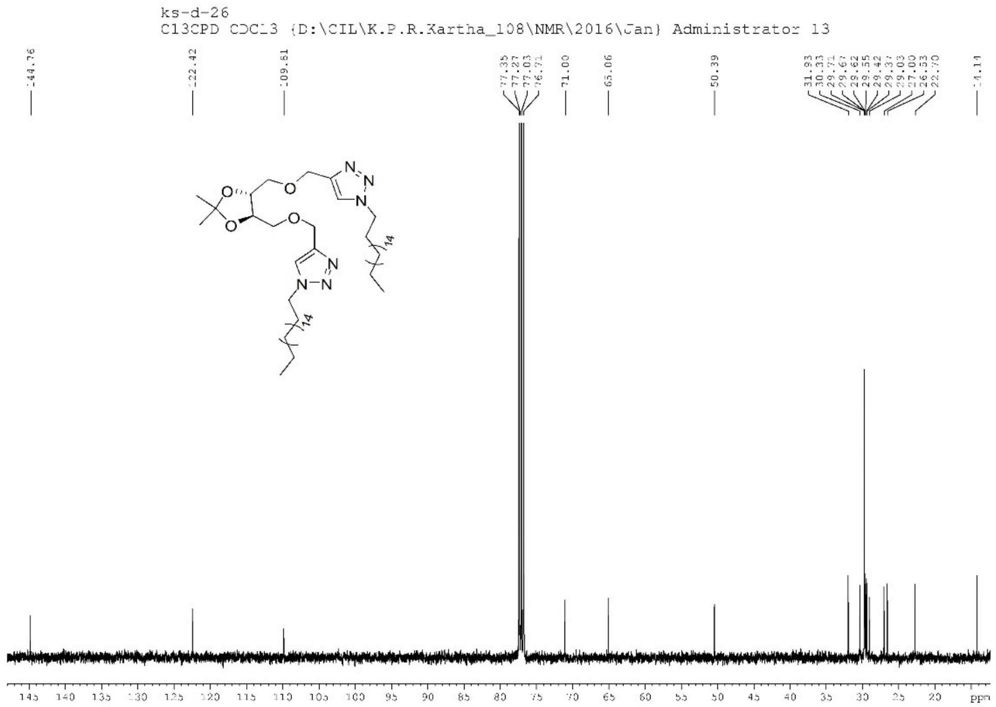

Supplement: RA-009-C9RA02868D-s001 [file RA-009-C9RA02868D-s001.pdf]
